# Optimally controlled nuclear magnetic resonance (NMR) in electrochemistry: Larmor versus nutation frequency selective spin excitation for locally selective NMR experiments

**DOI:** 10.5194/mr-7-113-2026

**Published:** 2026-07-17

**Authors:** Johannes F. Kochs, Armin J. Römer, Michael Schatz, Matthias Streun, Sven Jovanovic, Rüdiger-A. Eichel, Simone S. Köcher, Josef Granwehr

**Affiliations:** 1 Forschungszentrum Jülich GmbH, Institute of Energy Technologies, Fundamental Electrochemistry (IET-1), Jülich, Germany; 2 Institute of Technical and Macromolecular Chemistry, RWTH Aachen University, Aachen, Germany; 3 Forschungszentrum Jülich GmbH, Institute of Technology and Engineering (ITE), Jülich, Germany; 4 Institute of Physical Chemistry, RWTH Aachen University, Aachen, Germany; 5 Faculty of Mechanical Engineering, RWTH Aachen University, Aachen, Germany; 6 Fritz Haber Institute of the Max Planck Society, Berlin, Germany

## Abstract

Spectroelectrochemical nuclear magnetic resonance (NMR) experiments are faced with numerous challenges originating from shielding effects and susceptibility gradients in samples, leading to inhomogeneities in the static magnetic fields $B_{0}$ and the radio frequency (rf) fields $B_{1}$ . Moreover, magnetic feedback caused by eddy currents in conductors can obstruct precise measurements. Previous works have shown that these eddy-current-induced magnetic field distortions can be accurately predicted by finite element method (FEM) simulations. In this work, we present a workflow combining FEM predictions with quantum optimal control (QOC) to tailor custom NMR pulses that exploit specific magnetic field distortions for selective excitation of affected sample regions. The desired selectivity was achieved using pattern pulses optimized for either a particular $B_{1}$ or Larmor frequency $\nu_{0}$ . Experimental validation was performed on a heterogeneous phantom consisting of two cavities filled with two spectroscopically distinguishable liquids, one between copper disks to mimic an electrochemical cell and one between polymer disks as a reference. An over 30-fold suppression of the reference resonance in between polymer compared to the resonance in between copper disks was achieved, demonstrating how QOC-tailored pulses can selectively address FEM-predicted $B_{1}$ distortions in the vicinity of electrical conductors to achieve spatial selectivity with simultaneous $\nu_{0}$ robustness. It was also demonstrated how QOC-tailored pulses can selectively excite specific $\nu_{0}$ despite $B_{0}$ distortions, which implies that difficulties with conventional solvent suppression techniques in electrochemical setups can be mitigated using the adjustable robustness of QOC-tailored pulses. The presented approach sets the stage for gradient-free, localized in operando NMR in electrochemistry and material sciences, with the prospect of surface selectivity down to the detection limit of the setup.

## 1 Introduction

Spectroelectrochemical methods offer valuable, non-invasive in situ and in operando insights into electrochemical transformation processes, such as electrolysis and electrocatalysis. More advanced techniques pave the way to study crystallographic (Bommarito et al., 1992; Huang et al., 2018) and optical (Williamson et al., 2003; Holtz et al., 2014) properties, concentrations (Corson et al., 2020; Jovanovic et al., 2021), chemical environments (Corson et al., 2020; Faid et al., 2020), and metal coordination (Lee et al., 2022) within operating electrochemical cells. A fitting cell design depending on the applied technique is important to optimize accessibility for spectroscopic investigations, i.e., thin-layer cells to minimize solvent and background signal (Zoski, 2007). Spectroelectrochemical nuclear magnetic resonance (NMR) investigations offer additional flexibility through customization of the employed pulse sequences for a given experiment. However, conducting reliable NMR experiments on entire electrochemical cell setups, yielding credible, informative results about electrochemical transformation processes, raises several challenges.

Firstly, incorporating an entire electrochemical setup in an NMR tube including several electrodes (working, counter, reference), liquid electrolyte, and current collectors while maintaining electric contact for applying a potential requires custom-designed setups. A large variety of cell setups have been designed for electrochemical applications, ranging from flow mode and batch mode cells in 5 mm NMR tubes (Richards and Evans, 1975; Jovanovic et al., 2021) to custom battery housings (Pecher et al., 2017; Wolff et al., 2025) and commercial coin cell NMR setups (Walder et al., 2021). Schatz et al. (2022) followed a compromise approach by assembling a flexible NMR setup that yielded reproducible results while simultaneously being easily replicable without demanding special equipment.

Secondly, electrochemical transformation processes are predominantly located at interfaces, and their effectiveness is defined by electrochemical reaction rates and reaction mechanisms, as well as adsorption processes (Hamann and Vielstich, 2005). Standard NMR lacks selectivity, and its sensitivity is strained with regard to detecting surface species, compounded further by a signal that is mostly dominated by solvent and bulk species. While using conventional solvent suppression sequences to minimize solvent signals (McKay, 2009) is challenging in the presence of electrically conductive electrode components, reasonable spatial selectivity was achieved by magnetic resonance imaging (MRI) pulse sequences based on magnetic field gradients for spatial encoding via frequency or phase (Schatz et al., 2023). However, MRI experiments are limited by a compromise between spatial, spectral, and temporal resolution.

Finally, electrochemical cells contain several metallic elements. Electrical conductors locally distort both the static $B_{0}$ and oscillating $B_{1}$ field, i.e., radio frequency (rf) field, that NMR relies on, which results in reduced resolution, non-quantitative results, and potential artifacts. The rf modulation impedes the effectiveness of established selective pulse sequences such as BURP, which are not optimized for systems with inherently distorted $B_{1}$ fields (Geen and Freeman, 1991). However, the local field distortions can be assessed qualitatively and quantitatively by numerical finite element method (FEM) simulations, which are crucial for successful, robust in operando NMR cell development. FEM-based investigations of $B_{0}$ and $B_{1}$ fields around electric conductors have correctly reproduced experimental findings of $B_{1}$ field distortions due to the metallic skin effect (Ilott and Jerschow, 2017; Mohammadi and Jerschow, 2019), as well as the dependence of $B_{1}$ distortions on the orientation of the conductor (Ilott et al., 2014; Vashaee et al., 2015; Jovanovic et al., 2021).

FEM simulations have also been utilized to validate and optimize uniform $B_{1}$ distribution within in operando cell setups to study proton exchange membrane (PEM) fuel cells (Zhang et al., 2011), as well as battery applications (Aguilera et al., 2021; Sanders et al., 2022), up to commercial coin cell scales (Walder et al., 2021). Most recently, Schatz et al. (2024) presented a workflow to integrate FEM simulations of $B_{0}$ and $B_{1}$ into in operando cell development.

To account for inhomogeneities of either $B_{0}$ , i.e., Larmor frequencies $\nu_{0}$ , or $B_{1}$ , i.e., nutation frequency $\nu_{1}$ , or both simultaneously, quantum optimal control (QOC) has established itself as a versatile NMR pulse design method, particularly after the emergence of computationally efficient, numerical QOC methods such as gradient ascent pulse engineering (GRAPE) (Khaneja et al., 2005). QOC has been used for customized pulse optimization, with the goal of achieving robust broadband excitation covering extended ranges of inhomogeneities (Kobzar et al., 2004; Kobzar et al., 2008; Kobzar et al., 2012), selection of specific quantum coherence states (Köcher et al., 2016), or selective excitation or suppression of certain $\nu_{0}$ – $\nu_{1}$ combinations (Kobzar et al., 2005). Ilott et al. (2014) have exploited the skin effect of metallic lithium, in this case the $B_{1}$ field attenuation and phase change originating from eddy currents, to achieve either selective excitation within the skin depth of the metal or a suppression of the metal signal.

In the present work, we combine FEM simulations and QOC pulse design to tailor rf pulses for selective excitation or suppression of NMR signals in the vicinity of metallic copper elements. We show that $\nu_{1}$ -robust QOC pulses can still perform $\nu_{0}$ -selective excitation and suppression in the presence of conductive cell components. Furthermore, instead of just compensating for inhomogeneities, we introduce a new experimental approach, where the characteristic $B_{1}$ distortions in the proximity of conductive interfaces are exploited by $\nu_{1}$ -selective, $\nu_{0}$ -robust QOC pulses to achieve spatial selectivity without the need for pulsed field gradients. The pulse performances are experimentally demonstrated on a test setup consisting of cavities between copper coins and between polymer coins with different solvents to mimic potential future applications in electrocatalysis with metal electrodes.

## 2 Experimental methods and simulations

### 2.1 Quantum optimal control pulse optimization

The QOC pulses were optimized using a Python implementation of the GRAPE algorithm (Khaneja et al., 2005) with numerical efficiency boosted by efficient spin control using analytical Lie algebraic derivatives (ESCALADE) (Foroozandeh and Singh, 2021; Goodwin and Vinding, 2023). The SciPy implementation of the limited-memory Broyden–Fletcher–Goldfarb–Shanno algorithm (L-BFGS-B) was chosen as optimization back-end (Broyden, 1970; Fletcher, 1970; Goldfarb, 1970; Shanno, 1970; Byrd et al., 1995; Zhu et al., 1997;  Virtanen et al., 2020). To facilitate selectivity with respect to $\nu_{0}$ and $\nu_{1}$ , we used the concept of pattern pulses, a special variant of ensemble QOC where pulse optimization runs simultaneously across a whole ensemble of spin systems, each corresponding to a different combination of effective $\nu_{0}$ and $\nu_{1}$ (Kobzar et al., 2005). By assigning a target spin state $\rho_{\mathrm{target}}$ , corresponding to excitation or suppression, to each ensemble element, excitation patterns are achieved. Each ensemble element yields a quality factor computed as Re〈ρtarget|ρfinal〉 , the real part of the scalar product between the final spin state $\rho_{\mathrm{final}}$ after applying the pulse and $\rho_{\mathrm{target}}$ . The total quality function optimized by L-BFGS-B was computed as the weighted average of the ensemble quality factors. If not specified otherwise, the ensemble elements were weighted equally. The total control amplitude was limited by a hard upper bound of 10 kHz . Robustness with respect to $B_{0}$ variations was incorporated by variation of the Larmor frequencies $\nu_{0}$ , whereas robustness with respect to $B_{1}$ inhomogeneities was incorporated by allowing linear scalings of the nutation frequencies $\nu_{1}$ . The arising 2D grid of varying parameters is termed the excitation profile. The excitation profile resolution was set to 41 $\nu_{1}$ scalings times 401 $\nu_{0}$ offsets. The respective linear relation between $B_{0}$ and $\nu_{0}$ , as well as between $B_{1}$ and $\nu_{1}$ (Hore, 2015), is given by

1 $$\nu =-\frac{\gamma B}{2\pi }.$$

In this work, $B_{0}$ and $B_{1}$ in the vicinity of different materials are determined by FEM simulations. The B and ν terms are used interchangeably in the following unless an unambiguous distinction is required and stated. It must be noted that, instead of $B_{0}$ , the effective magnetic field that acts on a nuclear spin is used in this paper. That includes chemical (de-)shielding effects affecting the nucleus, as well as susceptibility effects of any material in its environment. Thus, $B_{0}$ , as used in this paper, may differ from the external, static magnetic field.

For the optimization of a $\nu_{0}$ -selective, $\nu_{1}$ -robust excitation pulse, the excitation profile was chosen such that spins within a $\nu_{0}$ range between -500 and 500 Hz are excited with $\rho_{\mathrm{target}}=I_{x}$ , whereas the remaining spins within the $\nu_{0}$ range from -2 to 2 kHz are suppressed, corresponding to $\rho_{\mathrm{target}}=I_{z}$ . Offsets outside of the selected bandwidth were not controlled. For a $\nu_{0}$ -selective suppression pulse, the target states were swapped. The linear scaling factors of $\nu_{1}$ ranged between 0.9 and 1.6. For the excitation pulse, a duration of 1 ms with 2000 equidistant time increments of 0.5 µs was sufficient to achieve a final mean quality factor of 94.9 %. For the suppression pulses, two distinct parameter sets were utilized: a duration of 1 ms with 2000 equidistant time increments of 0.5 µs (mean quality factor of 93.5 %) and a duration of 2 ms with 4000 equidistant time increments of 0.5 µs (mean quality factor of 95.6 %) to achieve a better frequency selectivity.

In the case of the suppression pulse, the quality factors of ensemble elements corresponding to a $\nu_{0}$ between -250 and 250 Hz were weighted by a factor of 5 relative to the remaining fidelities. Furthermore, the quality factors of ensemble elements corresponding to a $\nu_{1}$ scaling between 0.9 and 1.1 were additionally weighted by a factor of 5. This was due to the fact that the ensemble elements associated with these regions of the excitation profile tended to reach insufficient quality factor values for a homogeneous signal suppression. The pulse shapes and excitation profiles of the $\nu_{0}$ -selective excitation and suppression pulses are visualized in Sects. S1.1 and S2.1 in the Supplement (Figs. S1 to S3 and S10 to S12), respectively.

The $\nu_{1}$ -selective, $\nu_{0}$ -robust pattern pulse excitation profiles were set up based on the increase in $B_{1}$ intensity around metallic elements. The increase was quantified by FEM simulations of the local $B_{1}$ field in the copper and polymer (represented by vacuum) cavities of the model setup following the procedure described in Schatz et al. (2024). The simulated geometry is visualized in Sect. S3 (Fig. S19). The predicted relative $B_{1}$ increase is then determined as the ratio

2 $$\Gamma_{B_{1}}=\frac{B_{1}(\mathrm{Cu})}{B_{1}(\mathrm{vacuum})}.$$

For a given $\Gamma_{B_{1}}$ , the excitation region in the profile is defined as the set of ensemble elements corresponding to $\Gamma_{B_{1}}\pm 0.1$ . Ensemble elements in the excitation region are assigned $\rho_{\mathrm{target}}=I_{x}$ , and the remaining elements are assigned $\rho_{\mathrm{target}}=I_{z}$ . The $\nu_{0}$ offset range was chosen to be from -1 to 1 kHz . The range of $\nu_{1}$ scalings was chosen to be from 0.9 to 1.6, except for the pulse for selective excitation at $\Gamma_{B_{1}}=1.8$ , where the maximum $\nu_{1}$ scaling was raised to 1.9. A pulse duration of 1 ms with 2000 equidistant time increments of 0.5 µs was sufficient for all $\Gamma_{B_{1}}$ . The obtained final quality factors are listed in Table 1. The pulse shapes and excitation profiles of the $\nu_{1}$ -selective excitation and suppression pulses are visualized in Sects. S1.2 and S2.2 (Figs. S4 to S9 and S13 to S18), respectively.

**Table 1:** Obtained final mean quality factors for the optimization of $\nu_{1}$ -selective excitation pulses.

$\Gamma_{B_{1}}$	Mean Re〈ρtarget|ρfinal〉
1.00	95.5 %
1.20	96.5 %
1.25	96.9 %
1.30	96.6 %
1.40	96.6 %
1.80	96.4 %

**Figure 1:**
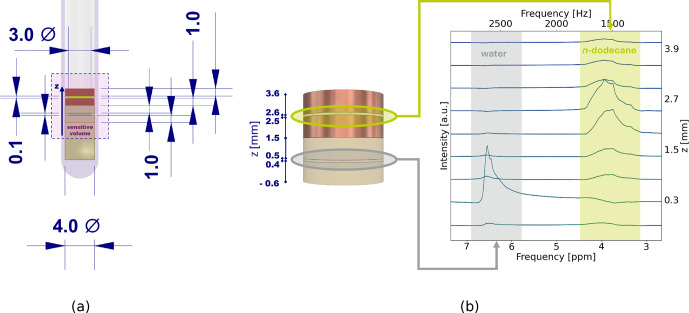
Sectional side view of experimental setup inside a shortened standard 5 mm NMR tube with cell dimensions given in mm **(a)** and 3D illustration of the setup with a spatially encoded chemical shift imaging of H_2_O and *n*-dodecane in between the double coins **(b)**. The cavities each have a diameter of 3 mm and a height of 0.1 mm . The coins each have a diameter of 4 mm and a height of 1 mm . The NMR-sensitive volume for homogeneous excitation is marked by a pink rectangle with dashed line and extends ±4 mm in the z direction from the center of the model setup. The spatial position of the liquids can be differentiated on a scale of about half a mm .

### 2.2 NMR experiments

A model setup was prepared to demonstrate the operating principle of the developed QOC pulses. To investigate the field-distorting effects of conductive materials on liquid samples, the setup contained two cavities of equal dimensions: the first one in between two copper coins and the second one in between two polymer coins made of polyether ether ketone (PEEK), serving as the reference cavity with minor magnetic field distortions. The rotation axes of both cylindrical cavities and their coins were oriented parallel to the $B_{0}$ field and perpendicular to the $B_{1}$ field, in accordance with the findings in a previous publication (Schatz et al., 2023). The model setup was fitted into a shortened common 5 mm NMR tube and is illustrated in Fig. 1a and b, including the diameter and thickness of copper and polymer coins, as well as the polymer spacers which defined the size of the cavity. The NMR tube was shortened to remove the narrowed opening such that the entire tube has a diameter of 4 mm .

Liquids were only added in between two coins of equal material, limiting the maximum filling height of liquid in each cavity to 0.1 mm . *n*-dodecane (in copper cavity) and H_2_O (in PEEK cavity) were chosen as non-mixable liquids, each of them filled into one of the two cavities, to enable an unambiguous distinction by spatial position, as well as by their chemical shift. The model setup was positioned in the NMR tube such that the two cavities were placed around the center of the NMR-sensitive volume.

A Bruker DiffBB BBO broadband diffusion probe and a Bruker Diff50 ^1^H diffusion probe, both with magnetic field gradients along the $B_{0}$ direction ( z axis) with a maximum gradient strength of 2312 $G\;{\mathrm{cm}}^{-1}$ were operated on a Bruker Avance III HD spectrometer (Bruker BioSpin GmbH, Rheinstetten, Germany) with a 9.4 T wide-bore magnet.

The spatial position of the two liquids was verified by phase-encoded ^1^H chemical shift imaging (CSI) along the z axis (Fig. 1b). Phase-encoded ^1^H CSI was performed by using pulsed magnetic field gradients along z with a strength of ±37.62 $G\;{\mathrm{cm}}^{-1}$ . The field of view was set to 20 mm with 32 points in the spatial dimension, resulting in a spatial resolution of 625 µm . Due to sharp susceptibility changes at interfaces, homogeneous shimming over the sample volume was not possible. Instead, the shims were adjusted for more pronounced separation of the resonances of the two compounds. No chemical shift reference was integrated into the model setup. Thus, the chemical shifts of water and *n*-dodecane do not correspond to their tabulated values.

Standard FID (free induction decay)-detected NMR experiments using a single hard pulse adjusted for a Bruker reference sample were compared to experiments where the hard pulse was substituted by QOC pulses. For the $\nu_{0}$ -selective QOC pulses, in order to illustrate the selective bandwidth of 1000 Hz over the total pulse bandwidth of 4000 Hz , the ^1^H resonance frequency offset $\Delta \nu_{0}$ was varied in steps of either 200 Hz or 300 Hz , starting 600 or 900 Hz downfield of the H_2_O resonance for a total frequency range of 2200 Hz or 2700 Hz , respectively. For the $\nu_{1}$ -selective pulses, $\Delta \nu_{0}$ was set such that the total pulse bandwidth of 2000 Hz contained the resonances of both *n*-dodecane and H_2_O. However, it was found that the pulse length required for a 90° pulse in the presented model setup differs from a Bruker reference sample. The actual 90° pulse lengths for each cavity were determined by a nutation experiment, and the resonance integrals in all reference spectra were adjusted accordingly. The acquisition time was adjusted to record the full FID, and the recovery delay was set to allow for complete relaxation. The baseline was corrected by using a splines fit.

Nutation experiments were performed with rectangular pulses of varying pulse length and a constant pulse power of 1.5 W for the Diff50 probe and 3.8 W for the DiffBB probe. In total, 100 pulse lengths were screened, with a step size of 30 µs .

## 3 Results and discussion

QOC pattern pulse design is capable of providing either selectivity or robustness with respect to both $\nu_{0}$ and $\nu_{1}$ independently. Each variation is exploited for specific applications. Selectivity with respect to the Larmor frequency $\nu_{0}$ allows for selective measurement of specific spin species, which enables solvent suppression or the suppression of other dominating bulk signals to increase sensitivity with respect to minority spin species. In contrast, $\nu_{0}$ robustness facilitates quantitative, phase-stable measurements in the presence of $B_{0}$ inhomogeneities. Robustness with respect to the nutation frequency $\nu_{1}$ allows for the uniform, quantitative excitation within an electrochemical cell despite $B_{1}$ -distorting metallic components. $\nu_{1}$ selectivity may be utilized for spatial selectivity on $B_{1}$ -distorting metallic surfaces.

With electrochemical applications in mind, this work discusses the two following QOC pulse types: firstly, $\nu_{1}$ -robust, $\nu_{0}$ -selective pulses enable efficient solvent suppression even in the presence of $B_{1}$ -distorting electrical conductors. Secondly, we demonstrate $\nu_{0}$ -robust, $\nu_{1}$ -selective excitation in order to achieve spatial selectivity in proximity to electrical conductors independently of their $B_{0}$ -distorting effects.

**Figure 2:**
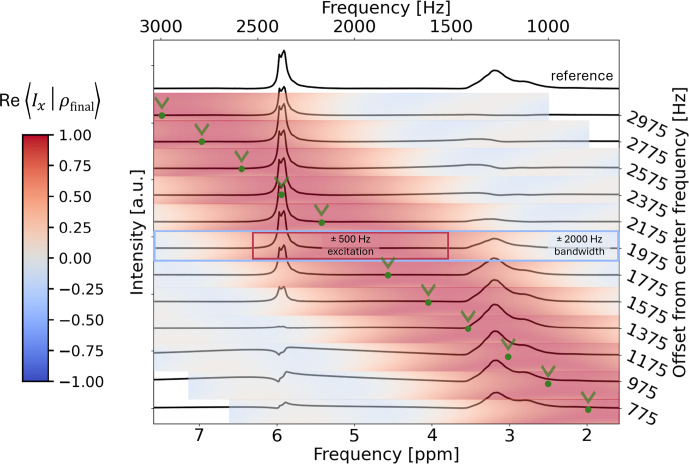
^1^H spectra recorded utilizing a $\nu_{0}$ -selective excitation pulse with a selective excitation range of 2.5 ppm ( ±500 Hz ), a 1 ms duration, and a total frequency range of 4000 Hz applied at different $\Delta \nu_{0}$ (marked with a green dot and arrow). The top spectrum depicts the reference ^1^H spectrum recorded using a hard pulse. Hereby, the resonance at approx. 3 ppm is assigned to *n*-dodecane, and the resonance at approx. 6 ppm is assigned to H_2_O. The spectra recorded with QOC pulses are underlaid with color gradients representing the theoretical x -magnetization Re〈Ix|ρfinal〉 , normalized to a range of [-1,1] , after applying the QOC pulse at each particular $\Delta \nu_{0}$ . Selective excitation is achieved for the on-resonance pulses with $\Delta \nu_{0}=2375$ Hz for H_2_O and $\Delta \nu_{0}=1375$ Hz for *n*-dodecane.

### 3.1 $\nu_{1}$ -robust $\nu_{0}$ Selectivity

The $\nu_{0}$ -selective QOC excitation pulses with a pulse length of 1 ms were demonstrated in a proof-of-concept experiment on the described model setup, with *n*-dodecane in the copper cavity and H_2_O in the PEEK cavity. Figure 2 illustrates the application of the $\nu_{0}$ -selective pulses at selected offsets $\Delta \nu_{0}$ . Here, $\Delta \nu_{0}$ was varied with a step size of 200 Hz between 2975 and 775 Hz (marked with a green dot and arrow), where the excitation is centered on either the resonance of H_2_O for $\Delta \nu_{0}=2375$ Hz and on the resonance of *n*-dodecane for $\Delta \nu_{0}=1375$ Hz . The top spectrum was recorded utilizing a hard pulse and serves as a reference for comparison. The linewidth of the H_2_O resonance (50 Hz ) is smaller compared to the coalesced *n*-dodecane resonance (150 Hz ). Both resonances show broad features due to a non-optimal shim caused by the significant magnetic susceptibility gradients throughout the model setup. Thus, the error on each individual resonance integral was calculated from the respective signal-to-noise ratio of each experiment.

When centering the excitation band on either resonance, the QOC pulse achieved efficient excitation, yielding a relative integral of 87.44±0.43 % for H_2_O or 115.34±0.26 % for *n*-dodecane compared to the 90° hard pulse. Simultaneously, the respective other resonance was suppressed to either 4.96±0.28 % or 4.02±0.40 % of its original value. The relative QOC integrals of both resonances compared to the reference for each of the $\nu_{0}$ -selective pulses are summarized in Table S1 in the Supplement.

**Figure 3:**
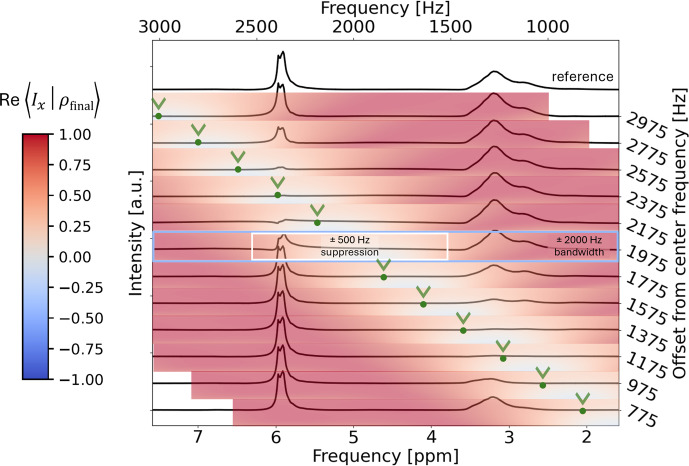
^1^H spectra recorded utilizing a $\nu_{0}$ -selective suppression pulse with a selective suppression range of 2.5 ppm ( ±500 Hz ), a 2 ms duration, and a total frequency range of 4000 Hz applied at different $\Delta \nu_{0}$ values. The top spectrum depicts the reference ^1^H spectrum recorded using a hard pulse. Hereby, the resonance at approx. 3 ppm is assigned to *n*-dodecane, and the resonance at approx. 6 ppm is assigned to H_2_O. The spectra recorded with QOC pulses are underlaid with color gradients representing the theoretical x -magnetization Re〈Ix|ρfinal〉 , normalized to a range of [-1,1] , after applying the QOC pulse at each particular $\Delta \nu_{0}$ . Selective suppression is achieved for the on-resonance pulses with $\Delta \nu_{0}=2375$ Hz for H_2_O and $\Delta \nu_{0}=1375$ Hz for *n*-dodecane.

Analogously, $\nu_{0}$ -selective QOC suppression pulses with the same pulse length were applied with a $\Delta \nu_{0}$ step size of 300 Hz . The pulses (Fig. S27 and Table S2) efficiently suppressed the H_2_O resonance to 7.42±0.97 % of its original value. However, the *n*-dodecane resonance was only suppressed to 32.66±0.32 %. This was due to an insufficient pulse performance at a duration of 1 ms , a non-optimal $\Delta \nu_{0}$ ( +100 Hz ) for *n*-dodecane in this specific experiment, and the large *n*-dodecane linewidth. To increase the suppression efficiency, the pulse duration was extended to 2 ms , and the step size was readjusted to 200 Hz such that the suppression band was centered on *n*-dodecane or H_2_O. In the adjusted experiment (Fig. 3 and Table S3), H_2_O was suppressed to 1.01±0.48 % and *n*-dodecane was suppressed to 1.29±0.26 % of their respective reference value when centering the suppression band on either of them, yielding a significantly improved suppression.

To assess the signal selectivity of the QOC excitation pulses quantitatively, a selectivity parameter

3 $$S_{\mathrm{QOC}}^{\mathrm{exc}}=\frac{I_{\mathrm{exc}}(I_{\sup,\mathrm{ref}}-I_{\sup})}{I_{\mathrm{exc},\mathrm{ref}}\;I_{\sup,\mathrm{ref}}}$$

was defined, where $I_{\mathrm{exc}}$ and $I_{\sup}$ denote the integrals of the excited and suppressed resonances obtained by the QOC pulse, respectively. Analogously, $I_{\mathrm{exc},\mathrm{ref}}$ and $I_{\sup,\mathrm{ref}}$ denote the integrals of the excited and suppressed resonances obtained by the hard 90° pulse, respectively. $S_{\mathrm{QOC}}$ is between 0 and 1 in the ideal case where the 90° hard-pulse reference achieves a uniform maximum excitation, where $S_{\mathrm{QOC}}=1$ describes an optimally selective and $S_{\mathrm{QOC}}=0$ a non-selective QOC excitation. However, QOC excitation surpassing the excitation of 90° hard pulses was observed experimentally, thus achieving $S_{\mathrm{QOC}}>1$ in some cases. We believe the origin of this effect lies within the capacitive interaction of the rf pulse $B_{1}$ and the conductive plates, which is more pronounced for the short, hard pulse than for the long, soft QOC pulse.

A corresponding selectivity parameter

4 $$S_{\mathrm{QOC}}^{\sup}=\frac{I_{\sup}(I_{\mathrm{exc},\mathrm{ref}}-I_{\mathrm{exc}})}{I_{\mathrm{exc},\mathrm{ref}}\;I_{\sup,\mathrm{ref}}}$$

was defined for QOC suppression pulses. In the following, $S_{\mathrm{QOC}}$ will be used to describe the selectivity of all QOC pulses and excitation, as well as suppression, referring to their respective $S_{\mathrm{QOC}}^{\mathrm{exc}}$ or $S_{\mathrm{QOC}}^{\sup}$ .

The highest $S_{\mathrm{QOC}}$ for $\nu_{0}$ -selective excitation in Fig. 2 amounted to 1.202±0.007 when positioning $\Delta \nu_{0}$ 200 Hz upfield from the center of the *n*-dodecane resonance ( $\Delta \nu_{0}=1175$ Hz , 11th spectrum from top). This was slightly higher compared to $S_{\mathrm{QOC}}=1.107\pm 0.006$ when the pulse was exactly on-resonance ( $\Delta \nu_{0}=1375$ Hz , 10th spectrum from top). Comparatively, selective suppression in Fig. 3 achieved a maximum $S_{\mathrm{QOC}}$ of 1.177±0.008 with $\Delta \nu_{0}$ 200 Hz upfield from the center of the *n*-dodecane resonance ( $\Delta \nu_{0}=975$ Hz, 12th spectrum from top), also slightly higher compared to $S_{\mathrm{QOC}}=1.096\pm 0.007$ when exactly on-resonance ( $\Delta \nu_{0}=1175$ Hz, 11th spectrum from top). Herein, excitation pulses achieved a similar maximum $S_{\mathrm{QOC}}$ as suppression pulses.

The achievable $S_{\mathrm{QOC}}$ in Fig. 3 was strongly affected by $\Delta \nu_{0}$ , even when within the pulse robustness range of ±500 Hz. For example, $S_{\mathrm{QOC}}$ is reduced to 0.397±0.004 and 0.839±0.006 when moving $\Delta \nu_{0}$ either downfield ( $\Delta \nu_{0}=1775$ Hz , 8th spectrum from top) or upfield ( $\Delta \nu_{0}=975$ Hz , 12th spectrum from top) by 400 Hz, respectively. $S_{\mathrm{QOC}}$ is also limited by the proximity of the suppressed and excited frequency ranges. The closer the resonance frequencies are to each other, the more challenging a selective excitation or suppression via $\nu_{0}$ -selective QOC pulses becomes, similarly to the case for conventional selective pulses (McKay, 2009). A near-instant transition from excitation to suppression along the $\nu_{0}$ dimension is difficult to realize in the pulse optimization and requires longer pulse durations for sharper transitions.

To overcome the selectivity difficulties for small frequency ranges, $B_{1}$ field variances originating from conductive materials were exploited instead of $\nu_{0}$ differences in the optimization of QOC pulses.

**Figure 4:**
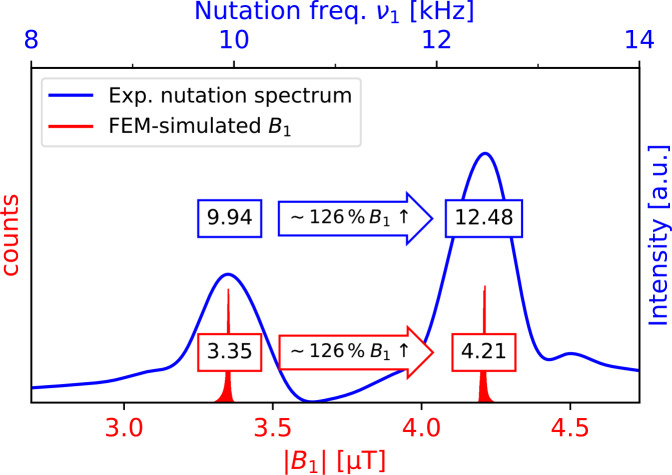
Dual x -axis plot superimposing the experimental nutation spectrum interpolated by a cubic spline (blue) and the FEM-simulated $B_{1}$ distribution (red). The nutation spectrum was recorded using rectangular pulses of varying pulse length at a constant pulse power (see Sect. 2.2). The FEM-simulated $B_{1}$ distribution was obtained as a histogram of the $B_{1}$ magnitudes at all of the finite-volume elements of the cavities. The smaller $\nu_{1}$ and $B_{1}$ values correspond to the PEEK cavity, while the larger $\nu_{1}$ and $B_{1}$ values correspond to the copper cavity. The relative difference between the $\nu_{1}$ maxima (25.6 %) is in good alignment with the relative $B_{1}$ increase predicted by the FEM simulation (25.7 %).

### 3.2 $\nu_{0}$ -robust $\nu_{1}$ selectivity

The FEM-predicted $B_{1}$ increase was validated experimentally via a nutation experiment (Fig. S20). A comparison of the obtained nutation frequencies $\nu_{1}$ for both cavities in the model setup is shown in Fig. 4. The FEM simulations revealed two narrow, clearly separated $B_{1}$ distributions for the copper and PEEK cavity with $\Gamma_{B_{1}}^{\mathrm{FEM}}=1.257$ . The experimentally determined $\Gamma_{B_{1}}^{\exp}$ amounted to 1.256, resulting in a difference of 0.001 between simulations and experiments. A difference of this magnitude is negligible when applying QOC pulses due to their robustness with respect to $B_{1}$ inhomogeneities of ±0.1 . Exchanging H_2_O by *n*-dodecane or other liquids affected neither the simulated nor the experimentally determined $\Gamma_{B_{1}}$ (Sect. S4.2.1). Thus, the difference in $B_{1}$ clearly originates from the surrounding material.

**Figure 5:**
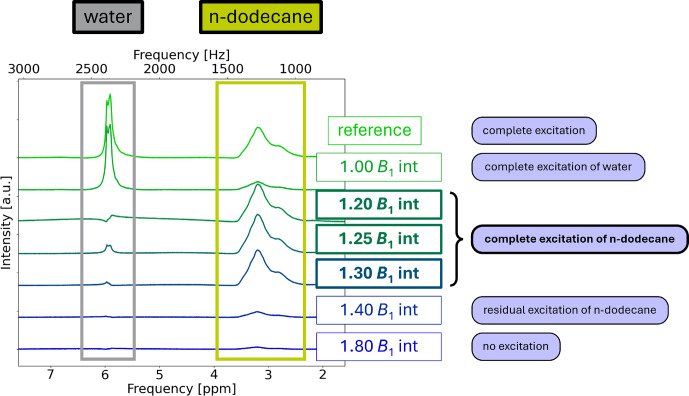
^1^H spectra recorded utilizing $\nu_{1}$ -selective excitation pulses with a selective excitation range of $\Gamma_{B_{1}}\pm 0.1$ , 1 ms duration and a total frequency range of 2000 Hz . Hereby, the resonance at approx. 3 ppm (framed in bright green) is assigned to *n*-dodecane, and the resonance at approx. 6 ppm (framed in gray) is assigned to H_2_O. The top spectrum depicts the reference ^1^H spectrum recorded using a hard pulse. Below, spectra utilizing selective QOC pulses, optimized for increasing $\Gamma_{B_{1}}$ , are displayed. Selective excitation of *n*-dodecane is achieved for $1.20\leq \Gamma_{B_{1}}\leq 1.30$ , which matches the $B_{1}$ amplification of the cavity in between copper.

To exemplify the accuracy of the method, a range of QOC pulses for varying $\Gamma_{B_{1}}$ with $\nu_{0}$ robustness (Sect. 2.1) was applied to the model setup and compared for a systematical screening of pulse effectiveness (Fig. 5). The errors of all values were calculated from the respective signal-to-noise ratio of each experiment.

A 90° hard pulse reference spectrum of the same setup was recorded for comparison. The relative excitations of both resonances compared to the reference for each of the $\nu_{1}$ -selective pulses are summarized in Table 2.

**Table 2:** Individual relative integral of the $\nu_{1}$ -selective QOC excitation pulses compared to a corresponding 90° hard pulse.

$\Gamma_{B_{1}}$	H_2_O excitation [%]	*n*-dodecane excitation [%]
1.00	99.79±0.49	16.53±0.30
1.20	7.76±0.64	90.06±0.40
1.25	11.57±0.60	106.25±0.37
1.30	3.10±0.56	96.19±0.46
1.40	0.73±0.53	14.09±0.33
1.80	1.11±0.55	3.63±0.34

The QOC pulses for $\Gamma_{B_{1}}=1.2$ , $\Gamma_{B_{1}}=1.25$ and $\Gamma_{B_{1}}=1.3$ selectively excited the *n*-dodecane resonance to a minimum of 90.06±0.49 % and simultaneously suppressed the resonance of H_2_O to a maximum of 11.57±0.60 %. These results confirm a successful QOC excitation of resonances inside the copper cavity while suppressing resonances inside the PEEK cavity within the $\Gamma_{B_{1}}$ robustness limit of ±0.1 . The highest excitation of *n*-dodecane was achieved with the pulse for $\Gamma_{B_{1}}=1.25$ , while the highest suppression of H_2_O was achieved with the pulse for $\Gamma_{B_{1}}=1.30$ .

In comparison, the application of a QOC pulse optimized for $\Gamma_{B_{1}}=1.0$ excited the H_2_O resonance to 99.79±0.49 % and suppressed the *n*-dodecane resonance to 16.53±0.30 % of their original values. This illustrates that, depending on the $\Gamma_{B_{1}}$ which the QOC pulses are optimized for, a resonance selectivity for either the copper cavity with increased $B_{1}$ or the PEEK cavity with no $B_{1}$ increase can be achieved. Furthermore, the additional spectra corresponding to even higher $\Gamma_{B_{1}}$ exemplify how a mismatch between the $\Gamma_{B_{1}}$ range of the QOC pulse and the $B_{1}$ experienced by both investigated spin systems leads to both resonances being suppressed to a maximum of 14.09±0.33 % ( $\Gamma_{B_{1}}=1.4$ ) and 3.63±0.34 % ( $\Gamma_{B_{1}}=1.8$ ) of their original values.

$S_{\mathrm{QOC}}$ was again determined to evaluate the effective signal selectivity of the $\nu_{1}$ -selective QOC pulses and to compare it to the $\nu_{0}$ -selective QOC pulses. Additionally, the experiments shown in Fig. 5 (except $\Gamma_{B_{1}}=1.25$ ) were each repeated three times for the determination of a standard deviation. Thus, the standard deviation will be used instead of the error based on the signal-to-noise ratio to evaluate the QOC pulse performance in the following. The average $S_{\mathrm{QOC}}$ values are summarized in Table 3.

**Table 3:** Average $S_{\mathrm{QOC}}$ of the $\nu_{1}$ -selective QOC excitation pulses. The corresponding 90° hard pulse has $S_{\mathrm{QOC}}=0$ .

$\Gamma_{B_{1}}$	$S_{\mathrm{QOC}}$
1.00	0.941±0.095
1.20	1.074±0.232
1.30	0.959±0.051
1.40	0.166±0.040
1.80	0.029±0.005

The highest average $S_{\mathrm{QOC}}$ was found for $\Gamma_{B_{1}}=1.2$ at 1.07±0.23 . For $\Gamma_{B_{1}}=1.3$ , the signal selectivity was, on average, slightly lower at $S_{\mathrm{QOC}}=0.96\pm 0.05$ . Comparing the theoretical $\Gamma_{B_{1}}$ of these pulses suggests that the pulse for $\Gamma_{B_{1}}=1.3$ should be more selective due to its $\Gamma_{B_{1}}$ being slightly closer to the experimentally determined value of 1.256. For the $\Gamma_{B_{1}}=1.2$ pulse, the experimental $\Gamma_{B_{1}}=1.256$ is therefore further away from the transition region of the QOC pulse, where excitation of the copper cavity transits to suppression of the PEEK cavity (Fig. S14) at 1.1 in contrast to the $\Gamma_{B_{1}}=1.3$ pulse with a transition at 1.2 (Fig. S16). However, the $S_{\mathrm{QOC}}$ deviation between the pulses for $\Gamma_{B_{1}}=1.2$ and $\Gamma_{B_{1}}=1.3$ lies in the range of the error bars. By comparison, QOC pulses for $\Gamma_{B_{1}}$ outside of the expected range result in a significantly lower average $S_{\mathrm{QOC}}$ of 0.166±0.040 ( $\Gamma_{B_{1}}=1.4$ ) and 0.029±0.005 ( $\Gamma_{B_{1}}=1.8$ ); thus, the selectivity parameter $S_{\mathrm{QOC}}$ precisely and reproducibly expresses the QOC pulse selectivity.

For completeness sake, we also tested the impacts of different rf pulse center frequencies, $B_{0}$ field shim, and receiver gain (Sect. S4.2.2 to S4.2.4) on the QOC spectra, which were revealed to be negligible, thus adding additional flexibility to the experimental implementation of QOC pulses.

## 4 Conclusions

In this work, a joint approach of $B_{1}$ simulation by FEM, numerical NMR pulse optimization by QOC, and the design of an electrochemically relevant model setup for in operando NMR was executed. QOC pulses that are $\nu_{1}$ -robust and $\nu_{0}$ -selective were able to selectively excite or suppress all resonances inside their selective $\nu_{0}$ bandwidth despite the presence of conductive cell components and the resulting $B_{1}$ distortions of the applied pulse. The $B_{0}$ distortions, which were evident from the line broadening of the *n*-dodecane resonances, also did not affect the results of the QOC experiments, demonstrating how QOC can be an effective tool to compensate for magnetic field distortions caused by conductive cell components. To support this claim, a comparison of QOC excitation pulses and E-BURP pulses (Geen and Freeman, 1991) based on the herein-presented model setup was undertaken (Fig. S28). The comparison evidently visualized that, when using literature-known $\nu_{0}$ -selective pulses without $\nu_{1}$ robustness for electrochemical setups, baseline distortions and unsatisfactory selectivity may occur due to strong $B_{1}$ field distortions near conductive cell components. Furthermore, the $B_{1}$ field distortions in the model setup were accurately predicted by FEM and integrated into a QOC workflow to tailor pattern pulses which exploit the simulated sharp $B_{1}$ enhancement near conductive interfaces. All spins which experienced the predicted $B_{1}$ increase were selectively and abundantly excited by suitable pattern pulses, while other spins were predominantly suppressed. While the $\nu_{0}$ -selective pulses targeted spin selectivity via the addressed Larmor frequency, the $\nu_{1}$ -selective pulses aimed for spatial selectivity based on the adjacent material. Both approaches achieved similar selectivity levels. Therefore, this study shows that magnetic field distortions are not just mere obstacles but can potentially be turned into exploitable features to tailor QOC pulses for different applications.

The selectivity parameter $S_{\mathrm{QOC}}$ was established to compare the performance of QOC pulses. Although QOC pulses differed in their performance, each of them clearly proved that this integrated approach can yield spatially selective data despite $B_{0}$ inhomogeneities and by taking advantage of strong $B_{1}$ field distortions. While selectivity can potentially be improved by increasing the duration of pattern pulses, it was shown that suitable contrast can be achieved with practically viable pulse durations on the order of 1 ms for real-world conditions, where surface relaxation or the presence of transient paramagnetic species may prevent the use of longer, more selective pulses.

The proof-of-concept experiments also revealed fundamental insights into conventional selective NMR pulses in conductive systems. While $B_{0}$ distortions near electrodes or metal components appear to be prominent in spectra, their extent is minor (ppm range) in comparison to the $B_{1}$ distortions (% range), validated by experiments and FEM simulations. Thus, $B_{1}$ distortions can be much more readily and effectively exploited for spatially selective QOC pulse optimization compared to the utilization of $\nu_{0}$ artifacts, which are also affected by chemical (de-)shielding effects.

Furthermore, this study also elucidates challenges of conventional solvent suppression methods in spectroelectrochemical NMR. The pulse sequences rely heavily on exact manipulation of the solvent magnetization, exploiting minute $\nu_{0}$ differences in the presence and absence of magnetic field gradients (Zheng and Price, 2010). With conductive materials, however, the large $B_{1}$ distortions disrupt the desired evolution of magnetization as the conventional suppression pulses yield divergent flip angles due to changed nutation frequencies. In addition, even small $B_{0}$ distortions near metallic surfaces may cause off-resonance effects. While robust suppression schemes, such as continuous-wave (CW) irradiation, may dismiss $B_{1}$ distortions, they typically have a too-narrow bandwidth to suppress susceptibility-broadened signals and may even heat up the volume in proximity to a metal electrode.

For future studies, the QOC pulse design can be adapted to currently relevant topics in electrochemistry, such as the formation of intermediates on the electrode surface during CO_2_ electrolysis or spatially selective investigations of the solid electrolyte interphase (SEI). Additionally, $\nu_{0}$ and $\nu_{1}$ selectivity of QOC pulses can be combined to achieve both spatially and chemically selective measurements at the same time. To facilitate spatial selectivity at a precisely controlled level, electrodes can also be customized with specific surface adjustments which result in distinct, easily predictable $B_{0}$ and $B_{1}$ distortions. In conclusion, the combination of electrochemical in operando NMR, FEM simulations, and QOC pulse optimization enables new experimental approaches with the potential to gain insights into local electrochemical phenomena that have previously been inaccessible and may help in answering complex research questions for which individual singular approaches might be insufficient. However, this workflow might be more difficult in the case of conductive materials with varying properties, such as porous electrodes or inhomogeneously distributed catalyst layers, requiring complex FEM simulations and exhibiting $B_{1}$ distortions that change during an experiment, leading to broad and transiently changing $B_{1}$ field distributions. Nevertheless, the presented method allows for the non-invasive and selective investigation of molecules near metal surfaces with high component flexibility and can take advantage of the wide nucleus range NMR has to offer.

## Supplement

10.5194/mr-7-113-2026-supplement The supplement related to this article is available online at https://doi.org/10.5194/mr-7-113-2026-supplement.

## Data Availability

TopSpin raw data of the presented measurements and pulse sequences are available with open access on the Jülich DATA repository at 10.26165/JUELICH-DATA/XQT0WN (Kochs et al., 2026). The simulation and optimization codes used in this work and all other data are available from the authors upon request.
