# Supplementary material for: Optimally controlled nuclear magnetic resonance (NMR) in electrochemistry: Larmor versus nutation frequency selective spin excitation for locally selective NMR experiments
Source: Magn Reson (Gott). 2026 Jul 17;7(2):113–23. doi: 10.5194/mr-7-113-2026 (PMC13430427; doi:10.5194/mr-7-113-2026)
Supplement: The supplement related to this article is available online at https://doi.org/10.5194/mr-7-113-2026-supplement. [file mr-7-113-2026-supplement.pdf]

Supplement of Magn. Reson., 7, 113–123, 2026
https://doi.org/10.5194/mr-7-113-2026-supplement
© Author(s) 2026. CC BY 4.0 License.

Open Access
MAGNETIC
RESONANCE

Supplement of

Optimally controlled nuclear magnetic resonance (NMR) in electrochemistry: Larmor versus nutation frequency selective spin excitation for locally selective NMR experiments

Johannes F. Kochs et al.

Correspondence to: Simone S. Köcher (s.koecher@fz-juelich.de)

The copyright of individual parts of the supplement might differ from the article licence.

Contents

S1 Pulse shapes of QOC pulses **2**
S1.1 Larmor frequency selective pulses 2
S1.2 Nutation frequency selective pulses 5

S2 Excitation Profiles **11**
S2.1 Larmor-frequency selective pulses 11
S2.2 Nutation-frequency selective pulses 13

S3 Experimental Setup **16**
S3.1 Setup in FEM simulation 16

S4 Supporting measurements **17**
S4.1 Nutation experiments 17
S4.2 Nutation frequency selective QOC pulse performance 18
S4.3 Larmor frequency selective QOC pulse performance 23

S5 Supporting data **25**
S5.1 Individual relative integrals of the B_0-selective QOC pulses 25

S1 Pulse shapes of QOC pulses

S1.1 Larmor frequency selective pulses

S1.1.1 ν_1-robust selective excitation within ±500 Hz in a ±2000 Hz suppression band

Figure S1 shows amplitude and phase of the pulse used for B_1-robust, selective excitation within ±500 Hz in a ±2000 Hz suppression band presented in Sec. 3.1 (Fig. 3) of the main article.

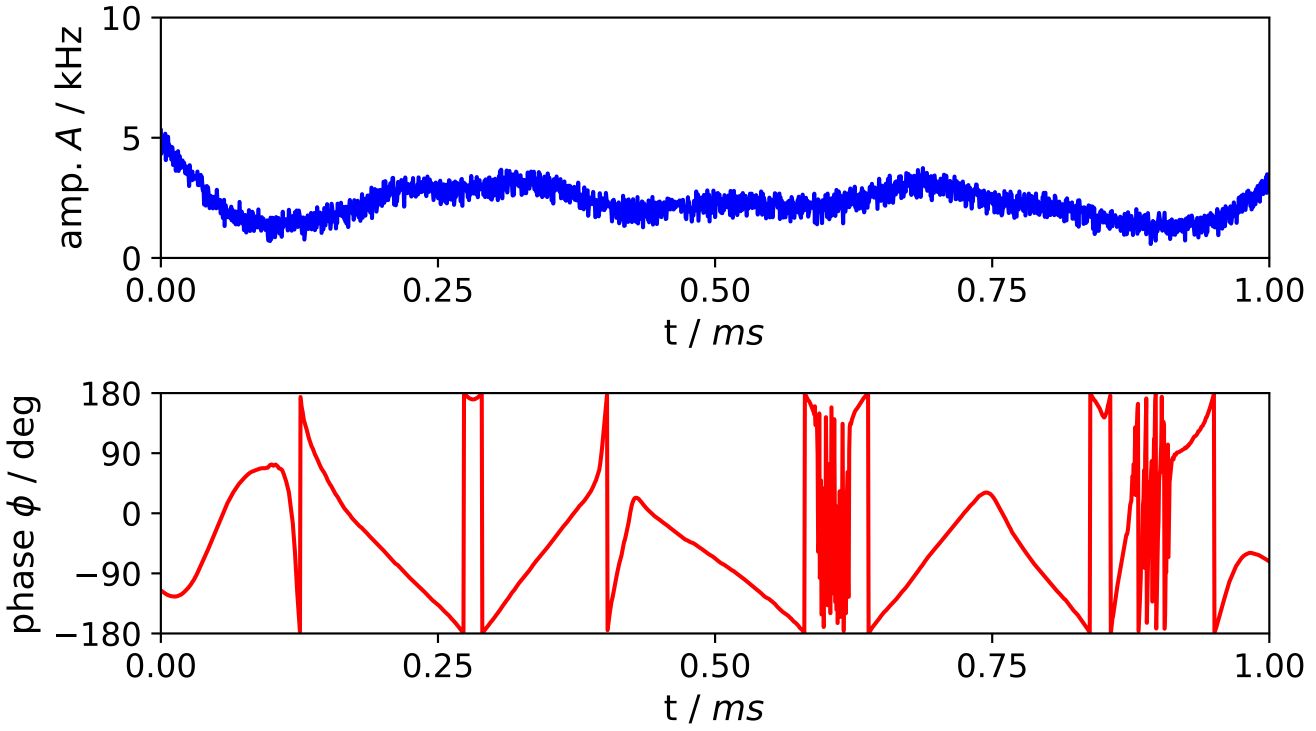

Figure S1. Pulse amplitude A and phase ϕ of the 1 ms Larmor-frequency selective excitation pulse.

S1.1.2 ν_1-robust selective suppression within ±500 Hz in a ±2000 Hz excitation band

Figure S2 and S3 below show amplitude and phase of the pulses used for B_1-robust, selective suppression within ±500 Hz in a ±2000 Hz excitation band presented in Sec. 3.1 of the main article. The experimental results in Sec. 3.1, Fig. 4 were obtained with the pulse in Fig. S3.

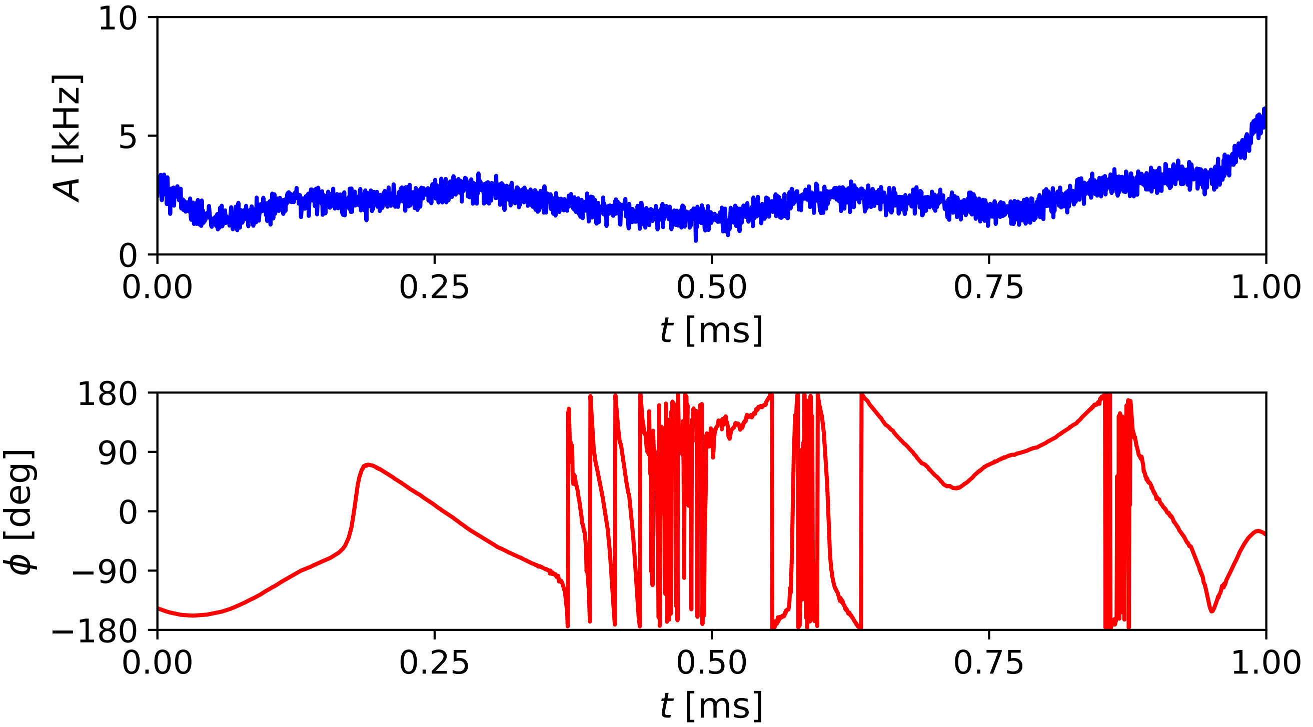

Figure S2. Pulse amplitude A and phase ϕ of the 1 ms Larmor-frequency selective suppression pulse.

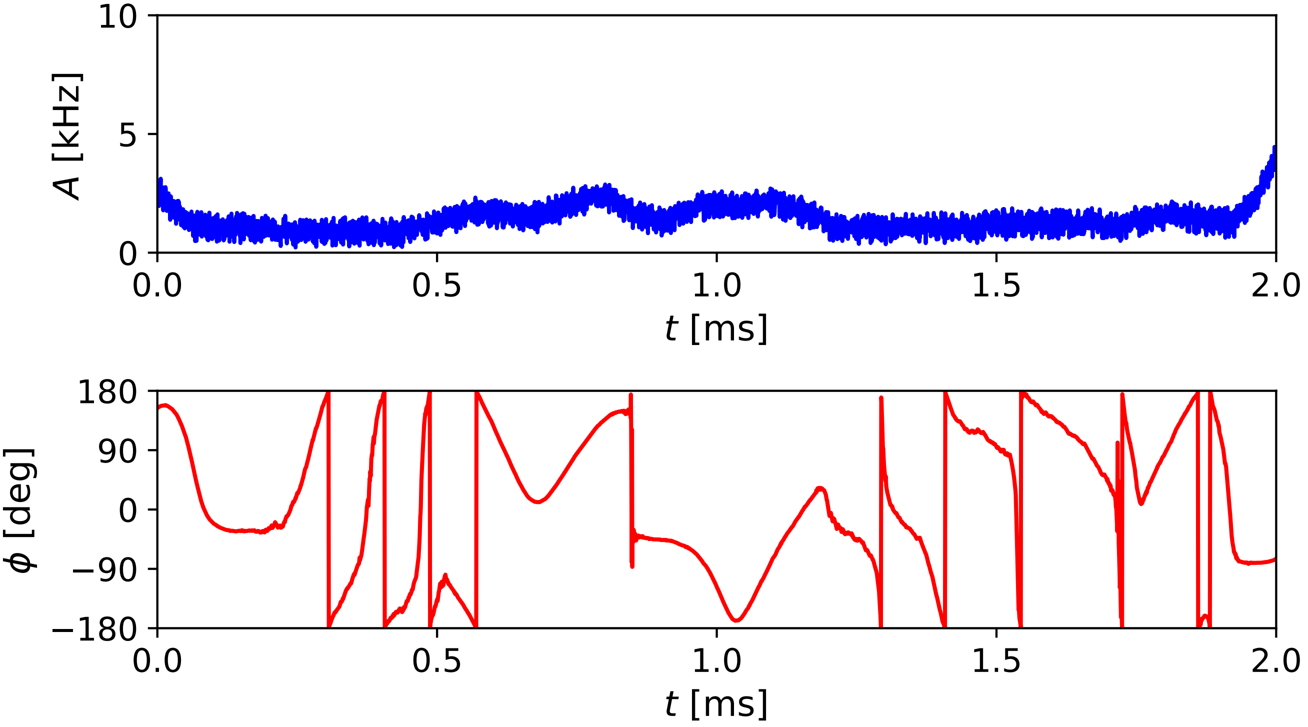

Figure S3. Pulse amplitude A and phase ϕ of the 2 ms Larmor-frequency selective suppression pulse.

S1.2 Nutation frequency selective pulses

The following Figures S4 to S9 show amplitude and phase of the pulses used for Larmor frequency-robust, ν_1-selective excitation for various artificial nutation frequency increases presented in Sec. 3.2 (Fig. 6) of the main article.

S1.2.1 ν_0-robust selective excitation for 0 % artificial nutation frequency increase

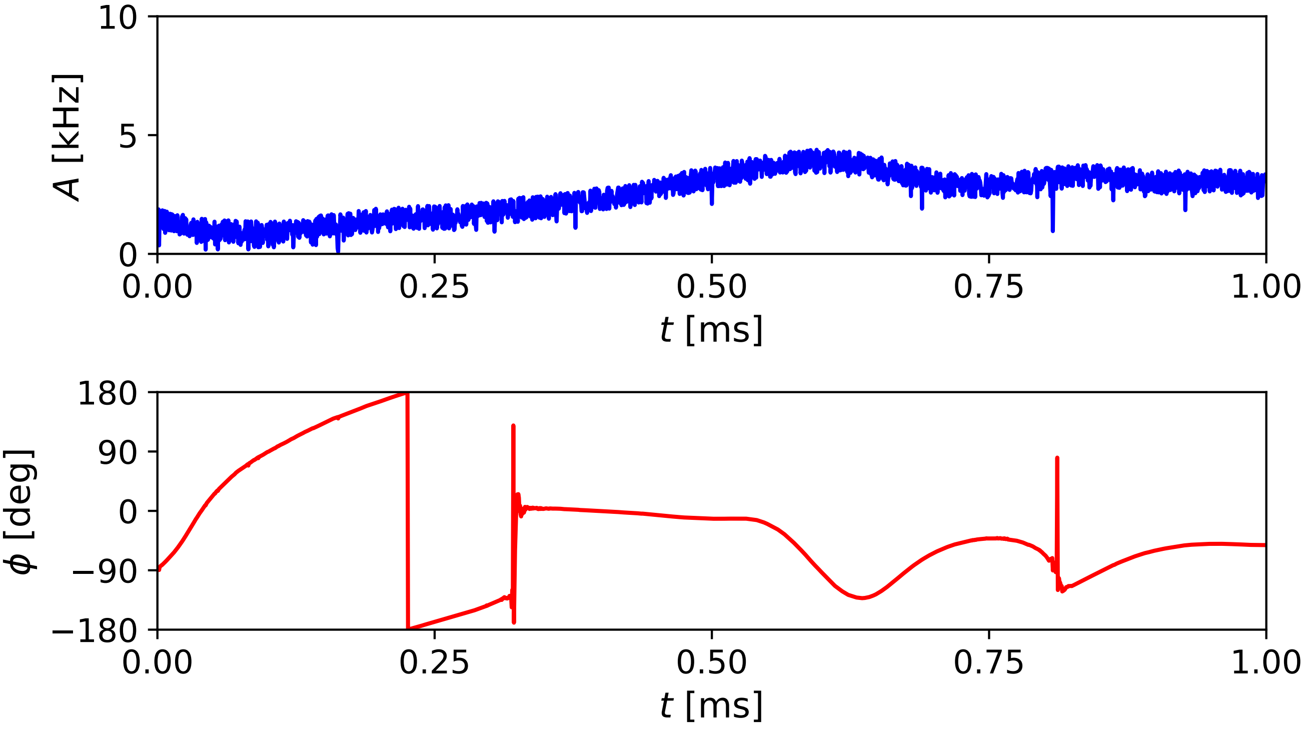

Figure S4. Pulse amplitude A and phase ϕ of nutation-frequency selective excitation for no B_1 increase.

S1.2.2 ν_0-robust selective excitation for 20 % artificial nutation frequency increase

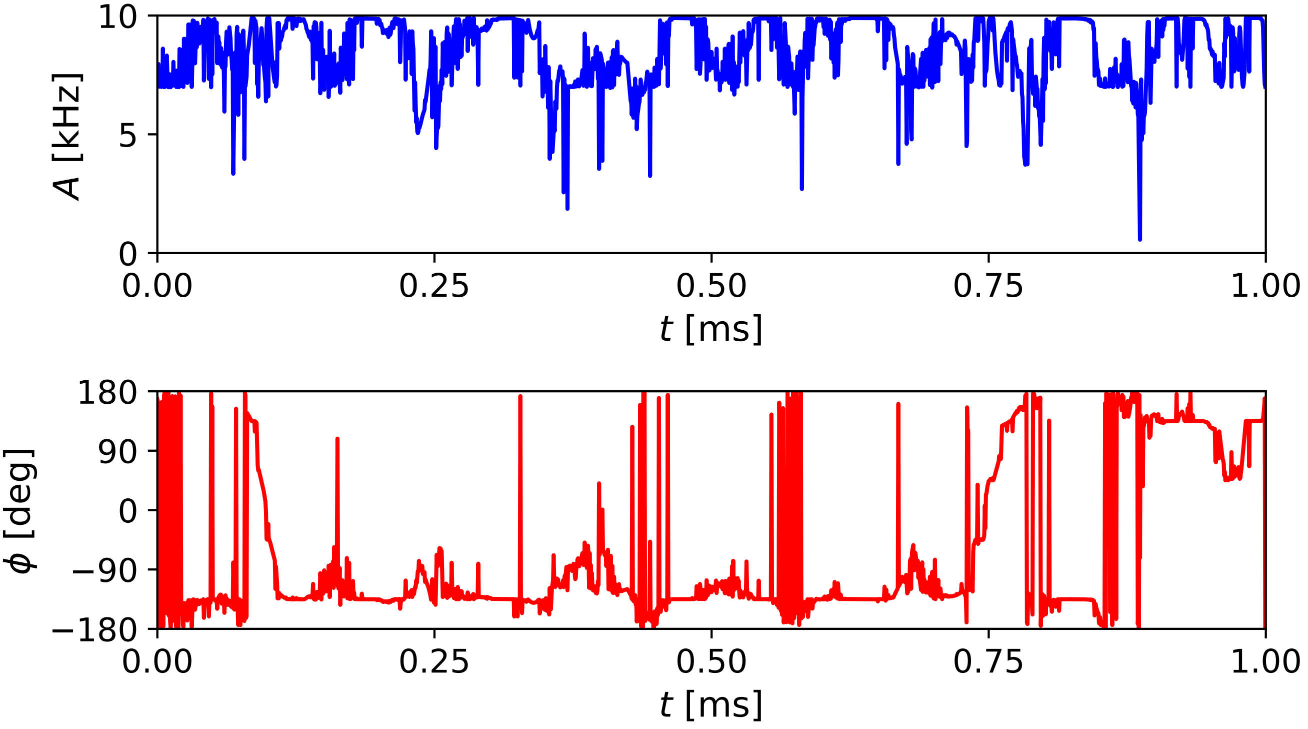

Figure S5. Pulse amplitude A and phase ϕ of nutation-frequency selective excitation for 20 % B_1 increase.

30 S1.2.3 ν_0-robust selective excitation for 25 % artificial nutation frequency increase

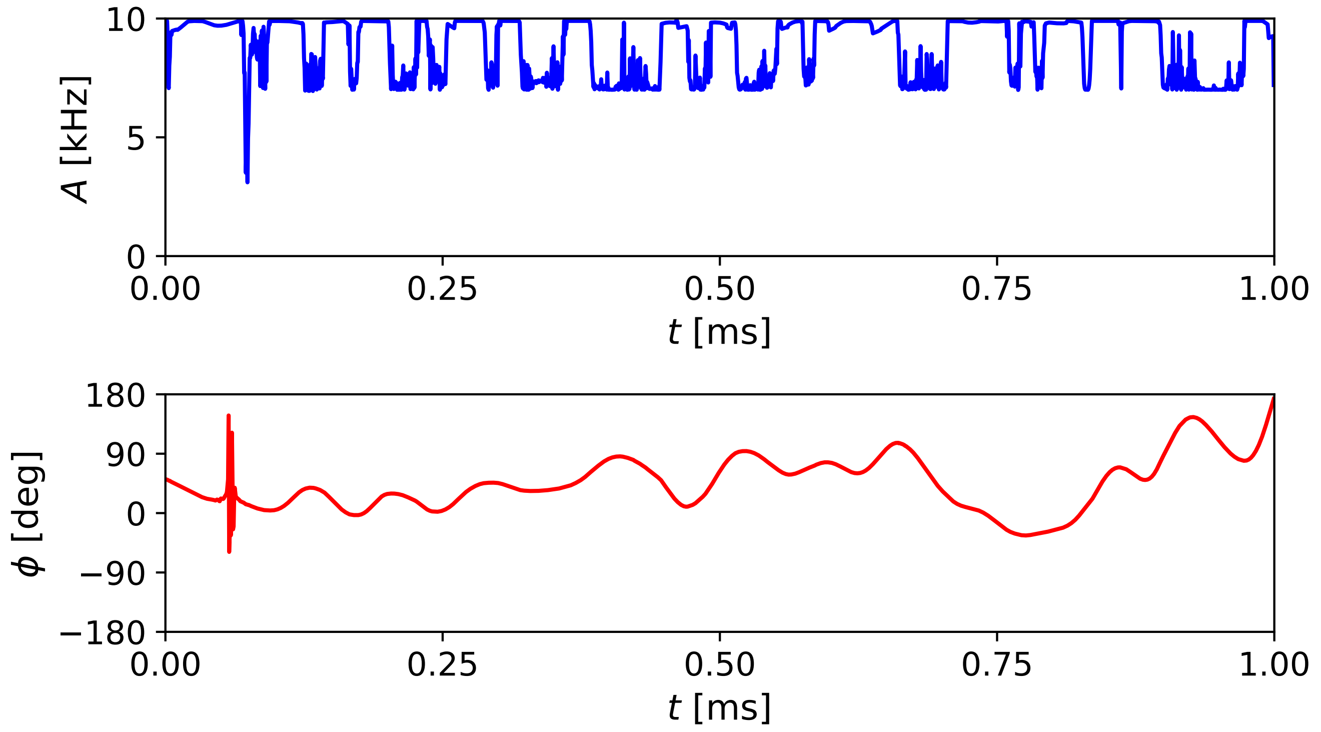

Figure S6. Pulse amplitude A and phase ϕ of nutation-frequency selective excitation for 25 % B_1 increase.

S1.2.4 ν_0-robust selective excitation for 30 % artificial nutation frequency increase

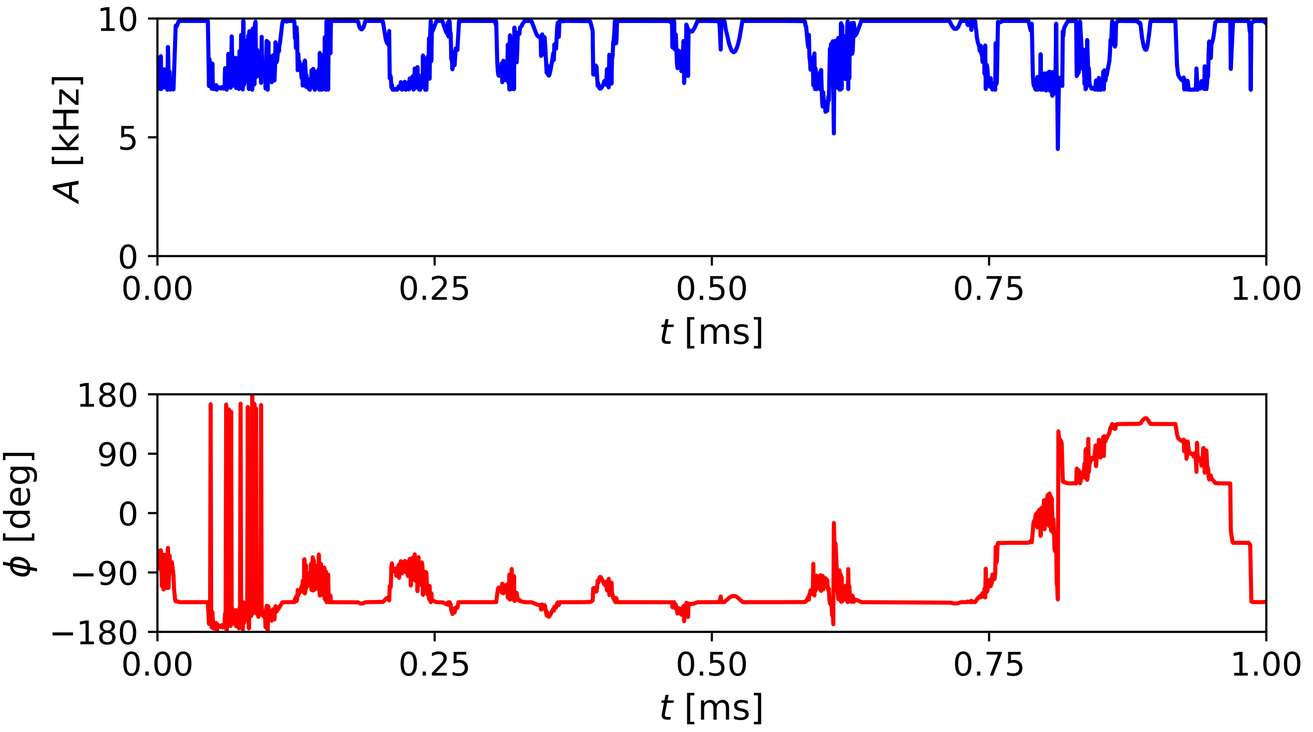

Figure S7. Pulse amplitude A and phase ϕ of nutation-frequency selective excitation for 30 % B_1 increase.

S1.2.5 ν_0-robust selective excitation for 40 % artificial nutation frequency increase

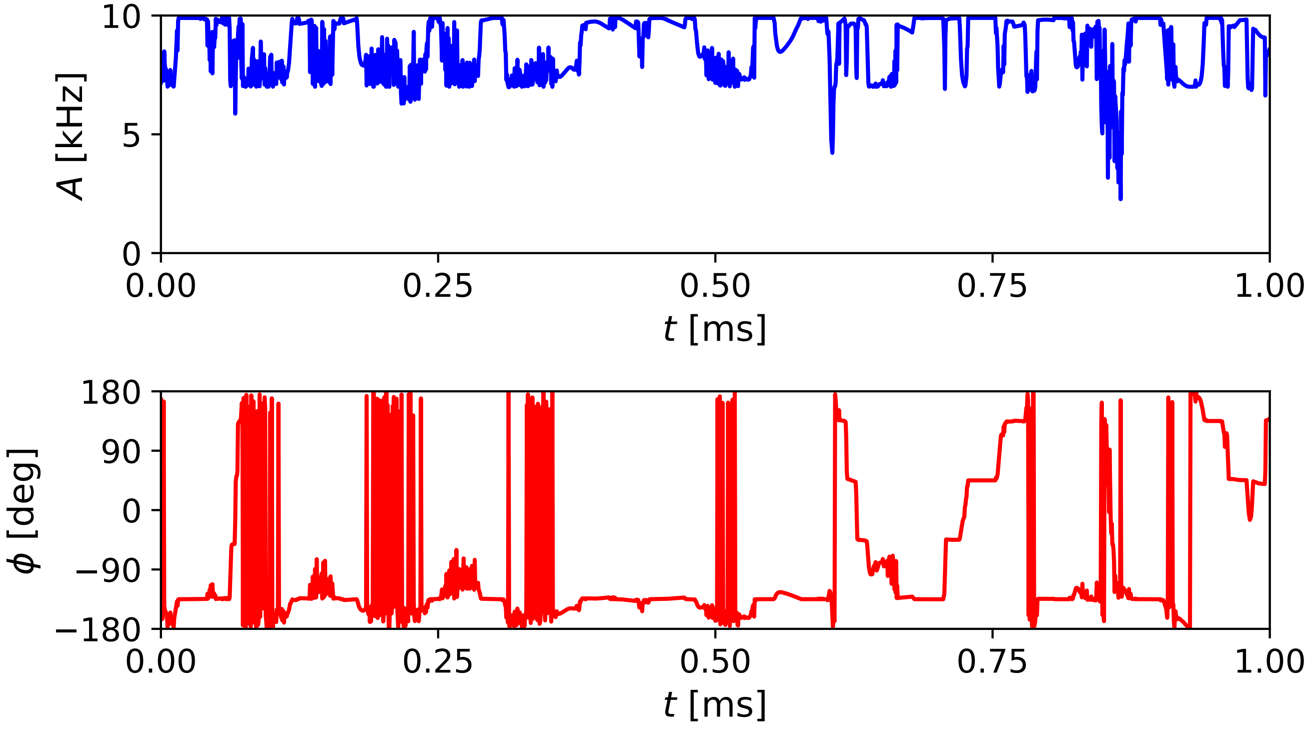

Figure S8. Pulse amplitude A and phase ϕ of nutation-frequency selective excitation for 40 % B_1 increase.

S1.2.6 ν_0-robust selective excitation for 80 % artificial nutation frequency increase

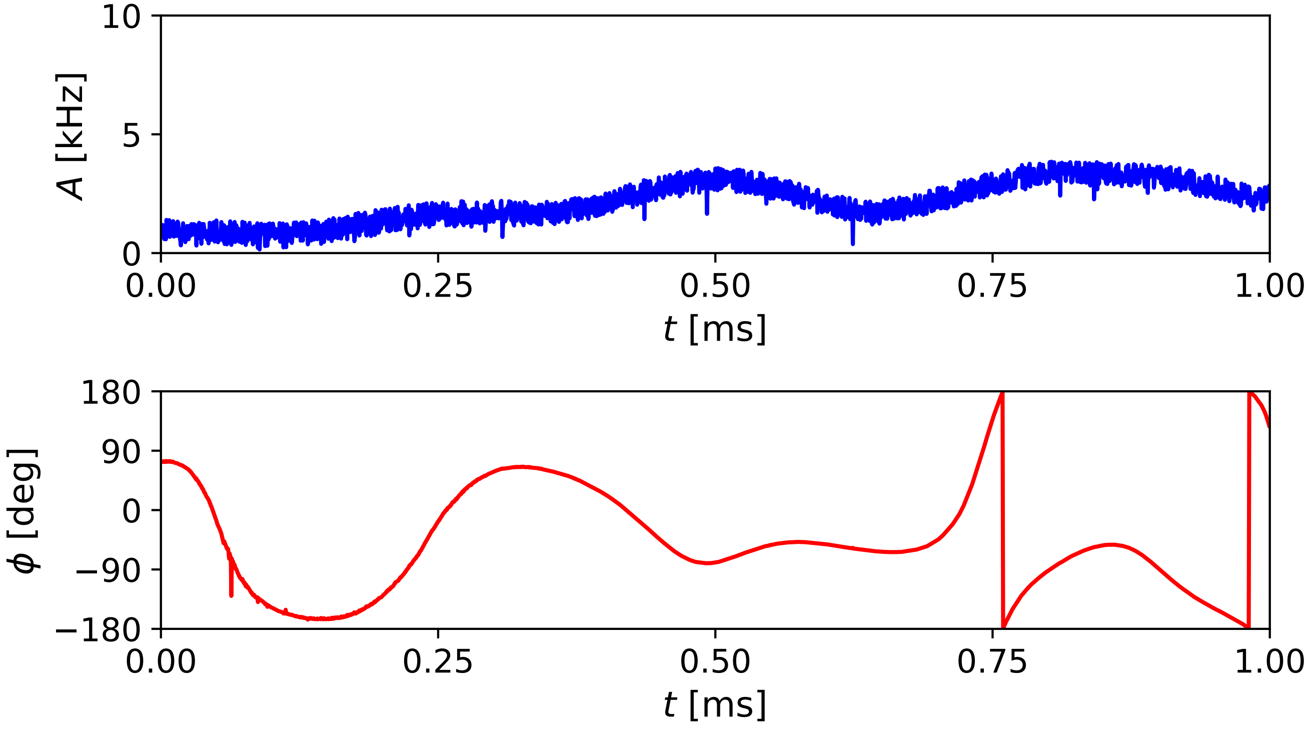

Figure S9. Pulse amplitude A and phase ϕ of nutation-frequency selective excitation for 80 % B_1 increase.

S2 Excitation Profiles

S2.1 Larmor-frequency selective pulses

S2.1.1 ν_1-robust selective excitation within ±500 Hz in a ±2000 Hz suppression band

Figure S10 shows the excitation profile of the pulse used for B_1-robust, selective excitation within ±500 Hz in a ±2000 Hz suppression band presented in Sec. 3.1 (Fig. 3) of the main article.

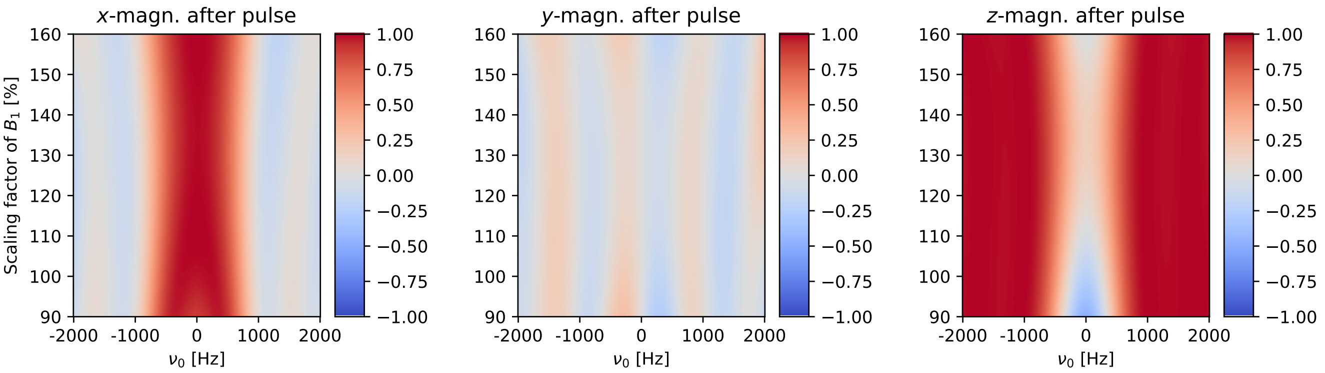

Figure S10. Simulated final x-, y- and z-magnetization for various Larmor-frequency offsets and B_1 increases after applying QOC pulse for Larmor-frequency selective excitation.

S2.1.2 ν_1-robust selective suppression within ±500 Hz in a ±2000 Hz excitation band

Figure S11 and S12 below show the excitation profiles of the pulses used for B_1-robust, selective suppression within ±500 Hz in a ±2000 Hz excitation band presented in Sec. 3.1 of the main article. The experimental results in Sec. 3.1, Fig. 4 were obtained with the pulse in Fig. S12.

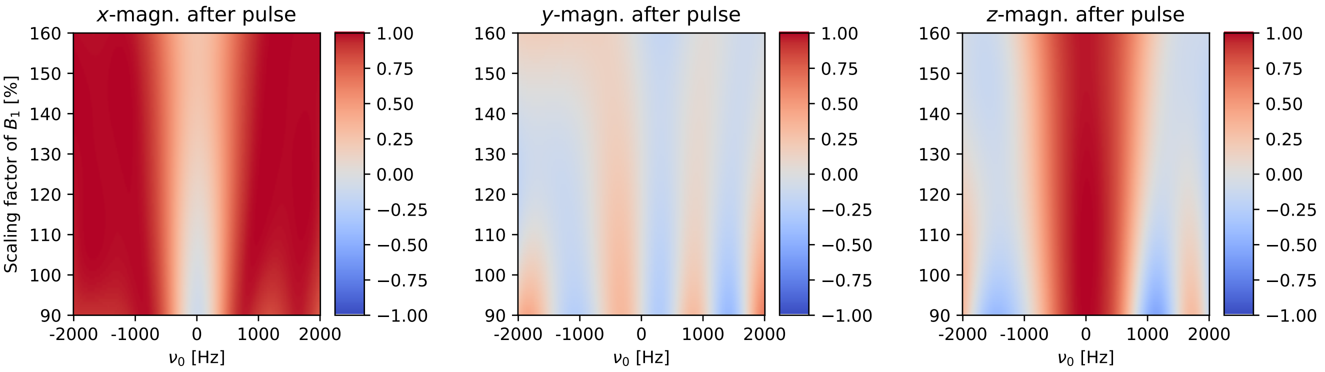

Figure S11. Simulated final x-, y- and z-magnetization for various Larmor-frequency offsets and B_1 increases after applying a 1 ms QOC pulse for Larmor-frequency selective suppression.

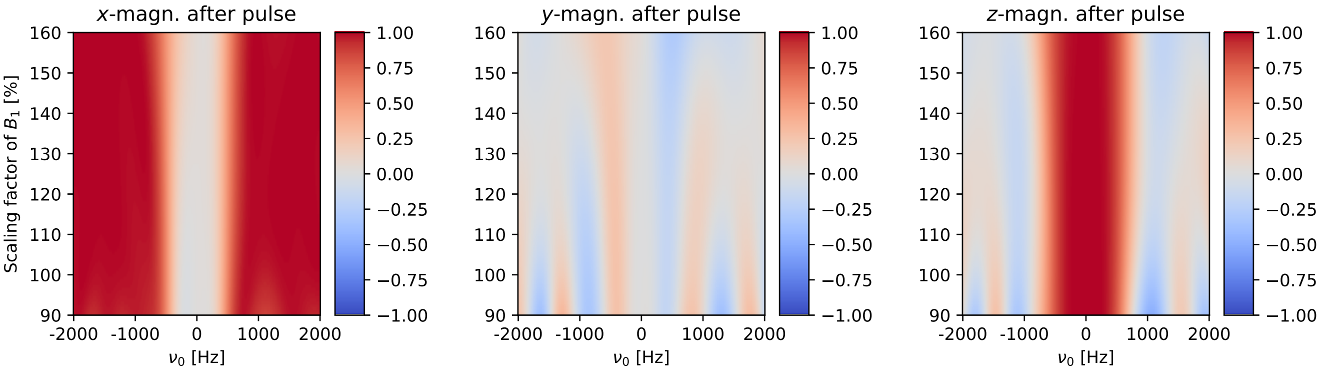

Figure S12. Simulated final x-, y- and z-magnetization for various Larmor-frequency offsets and B_1 increases after applying a 2 ms QOC pulse for Larmor-frequency selective suppression.

S2.2 Nutation-frequency selective pulses

The following Figures S13 to S18 show the excitation profiles of the pulses used for Larmor-frequency-robust, selective excitation for various artificial nutation frequency increases presented in Sec. 3.2 (Fig. 6) of the main article.

S2.2.1 ν_0-robust selective excitation for 0 % artificial nutation frequency increase

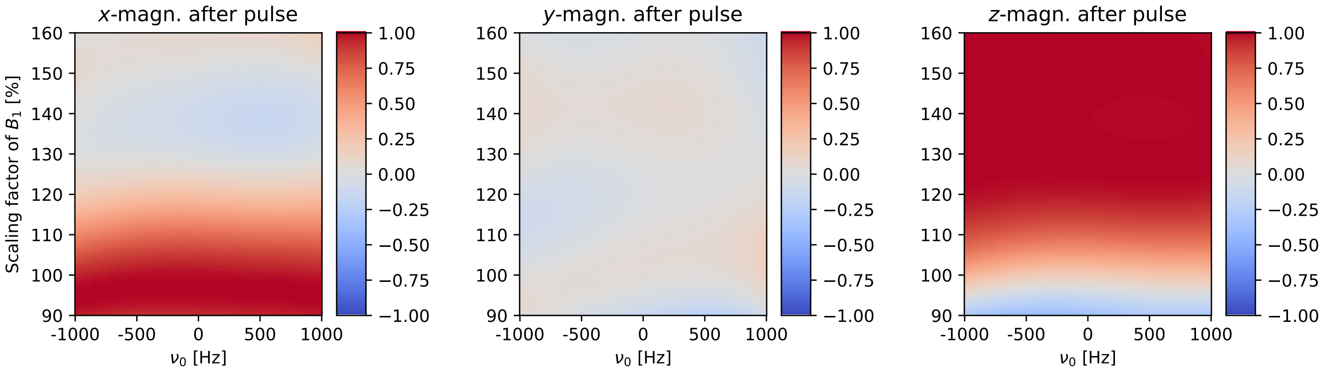

Figure S13. Simulated final x-, y- and z-magnetization for various Larmor-frequency offsets and B_1 increases after applying QOC pulse for nutation-frequency selective excitation for no B_1 increase.

S2.2.2 ν_0-robust selective excitation for 20 % artificial nutation frequency increase

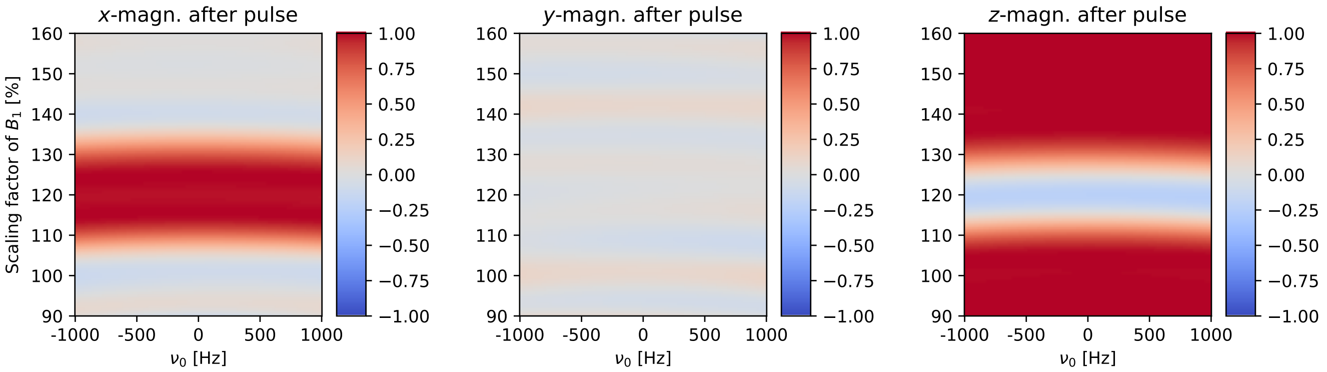

Figure S14. Simulated final x-, y- and z-magnetization for various Larmor-frequency offsets and B_1 increases after applying QOC pulse for nutation-frequency selective excitation for 20 % B_1 increase.

S2.2.3 ν_0-robust selective excitation for 25 % artificial nutation frequency increase

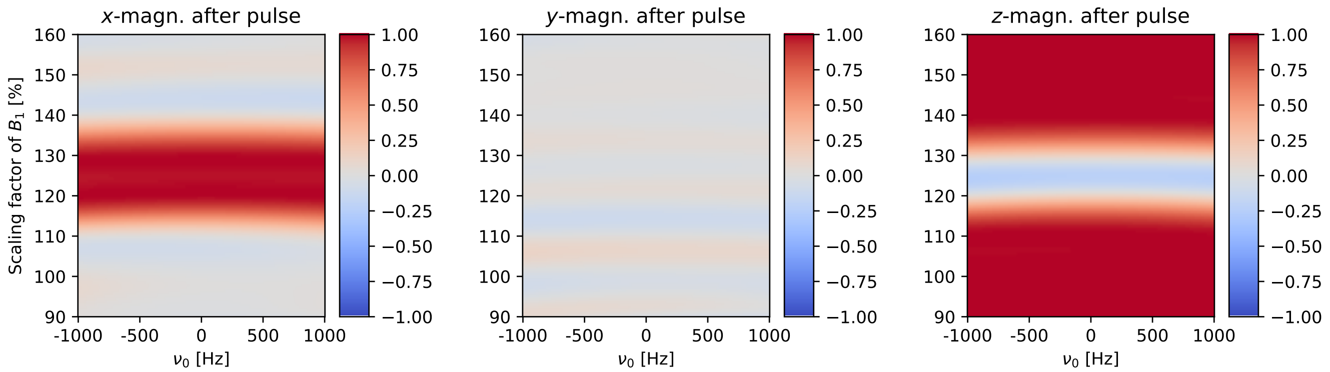

Figure S15. Simulated final x-, y- and z-magnetization for various Larmor-frequency offsets and B_1 increases after applying QOC pulse for nutation-frequency selective excitation for 25 % B_1 increase.

S2.2.4 ν_0-robust selective excitation for 30 % artificial nutation frequency increase

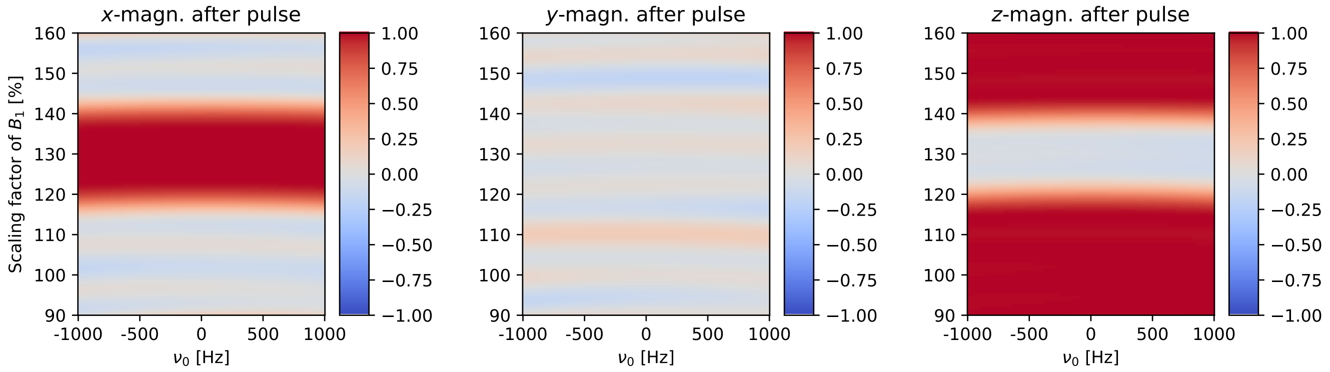

Figure S16. Simulated final x-, y- and z-magnetization for various Larmor-frequency offsets and B_1 increases after applying QOC pulse for nutation-frequency selective excitation for 30 % B_1 increase.

S2.2.5 ν_0-robust selective excitation for 40 % artificial nutation frequency increase

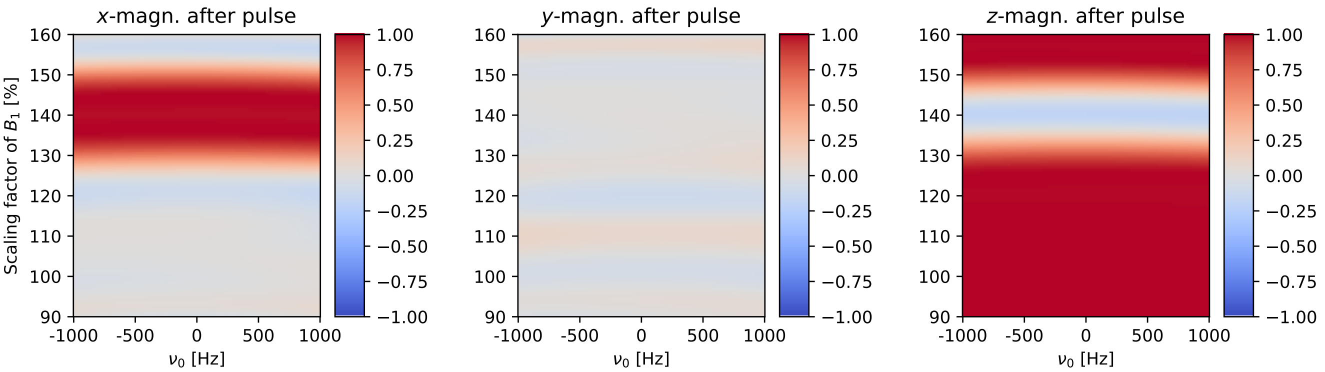

Figure S17. Simulated final x-, y- and z-magnetization for various Larmor-frequency offsets and B_1 increases after applying QOC pulse for nutation-frequency selective excitation for 40 % B_1 increase.

S2.2.6 ν_0-robust selective excitation for 80 % artificial nutation frequency increase

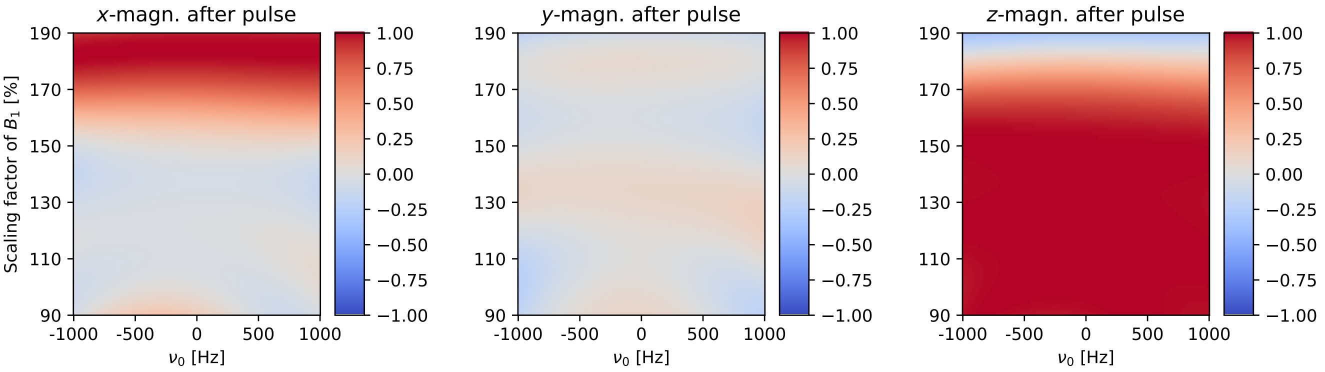

Figure S18. Simulated final x-, y- and z-magnetization for various Larmor-frequency offsets and B_1 increases after applying QOC pulse for nutation-frequency selective excitation for 80 % B_1 increase.

S3 Experimental Setup

S3.1 Setup in FEM simulation

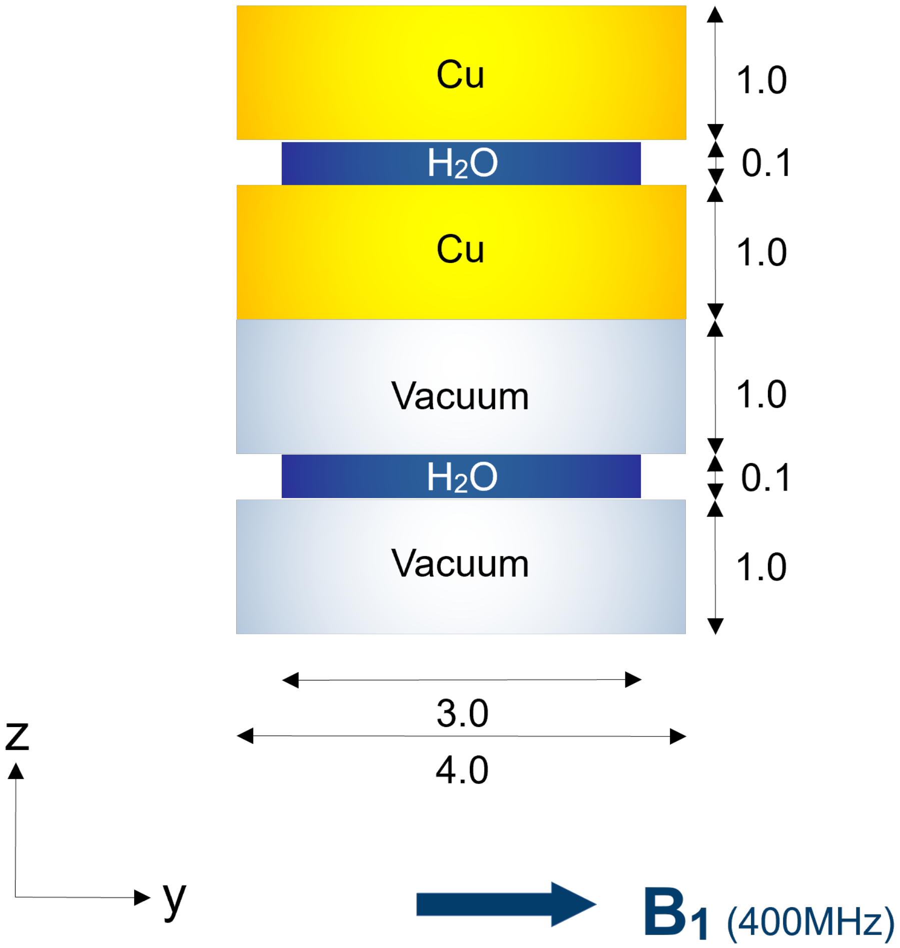

Figure S19. Cell parameters of the model setup in the FEM simulation. The volume between the polymer double coin was introduced as if in-between two cylindrical volumes of vacuum which have a conductivity of zero. Water was adopted as the corresponding liquid in both cavities, as the liquid composition does not affect Γ_{B_1}. The independence was verified by also simulating with n-dodecane as corresponding liquid for both cavities or only for the copper cavity.

S4 Supporting measurements

S4.1 Nutation experiments

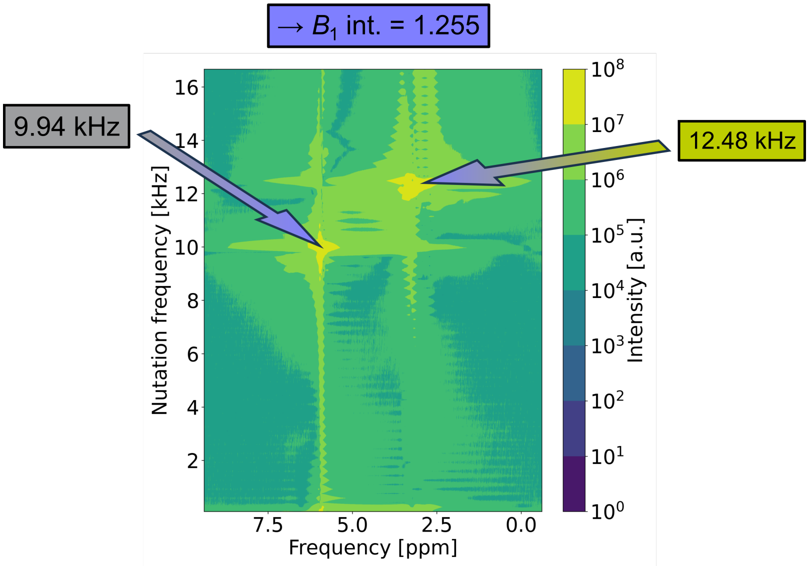

Figure S20. ^{1}H NMR 2D spectrum of the model setup, describing the intensity distribution of nutation frequencies in dependence of their respective Larmor frequency. The experimentally determined nutation frequency of H_2O is denoted in the grey box while the nutation frequency of *n*-dodecane is denoted in the bright green box. A color gradient from dark blue to bright green is used to illustrate the range from low to high signal intensities.

S4.2 Nutation frequency selective QOC pulse performance

S4.2.1 ν_1-selective QOC pulse performance dependence on the liquid composition

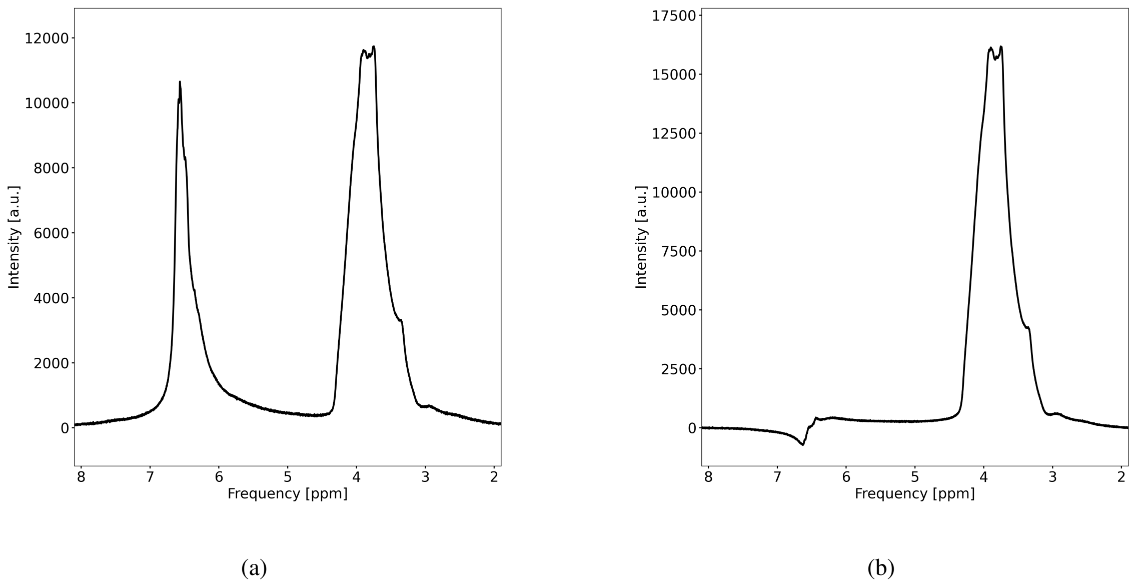

Figure S21. Comparison of the ^{1}H NMR spectrum of the model setup for a 90° hard pulse (a) to a QOC pulse optimized for $\Gamma_{B_1} = 1.2$ (b). Hereby, the resonance at approx. 3.8 ppm is assigned to *n*-dodecane inside the copper cavity while the signal at approx. 6.5 ppm is assigned to H_2O in the PEEK cavity.

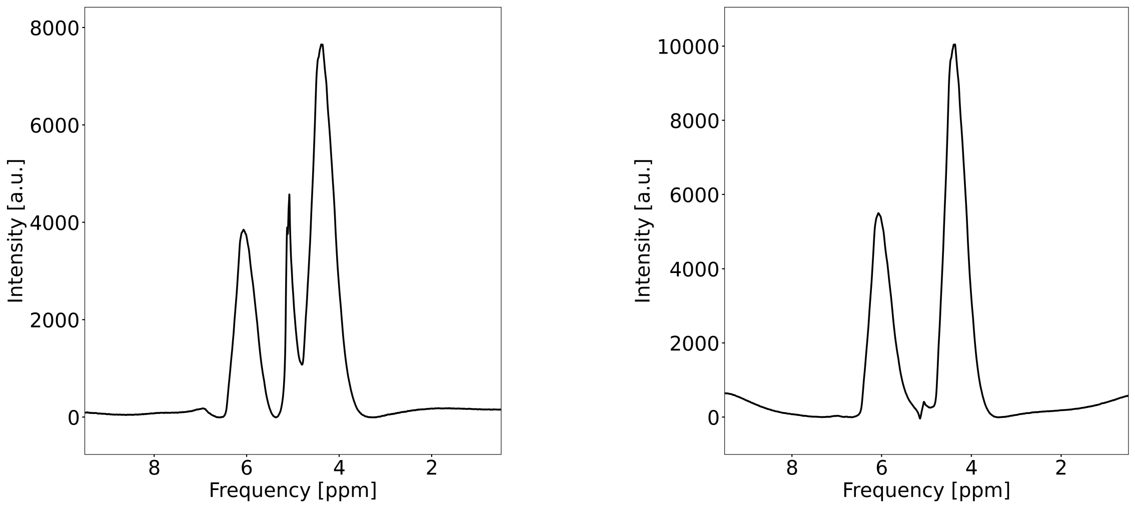

(a) (b)

Figure S22. Comparison of the ^{1}H NMR spectrum of the model setup for a 90° hard pulse (a) to a QOC pulse optimized for $\Gamma_{B_1} = 1.2$ (b). Hereby, the resonances at approx. 4.2 and 6 ppm are assigned to dimethylsulfoxide inside the copper cavity while the signal at approx. 5 ppm is assigned to H_2O in the PEEK cavity. The H_2O resonance is as effectively suppressed as in SI Fig. S21.

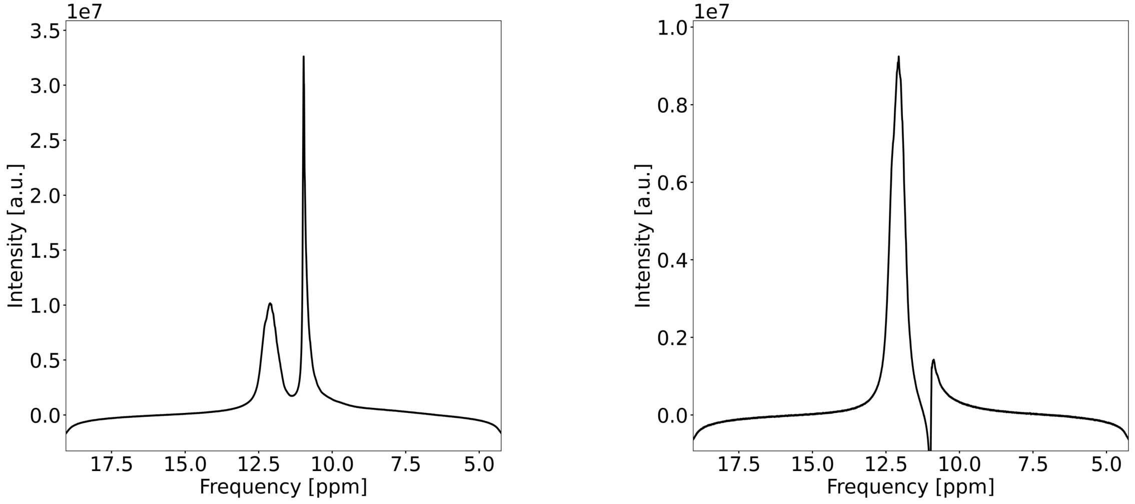

(a) (b)

Figure S23. Comparison of the ^{1}H NMR spectrum of the model setup for a 90° hard pulse (a) to a QOC pulse optimized for $\Gamma_{B_1} = 1.2$ (b). Hereby, the resonance at approx. 10.5 ppm is assigned to H_2O inside the copper cavity while the signal at approx. 12 ppm is assigned to H_2O in the PEEK cavity. The H_2O resonance in-between PEEK is as effectively suppressed as in SI Fig. S21.

S4.2.2 ν_1-selective QOC pulse performance dependence on receiver gain

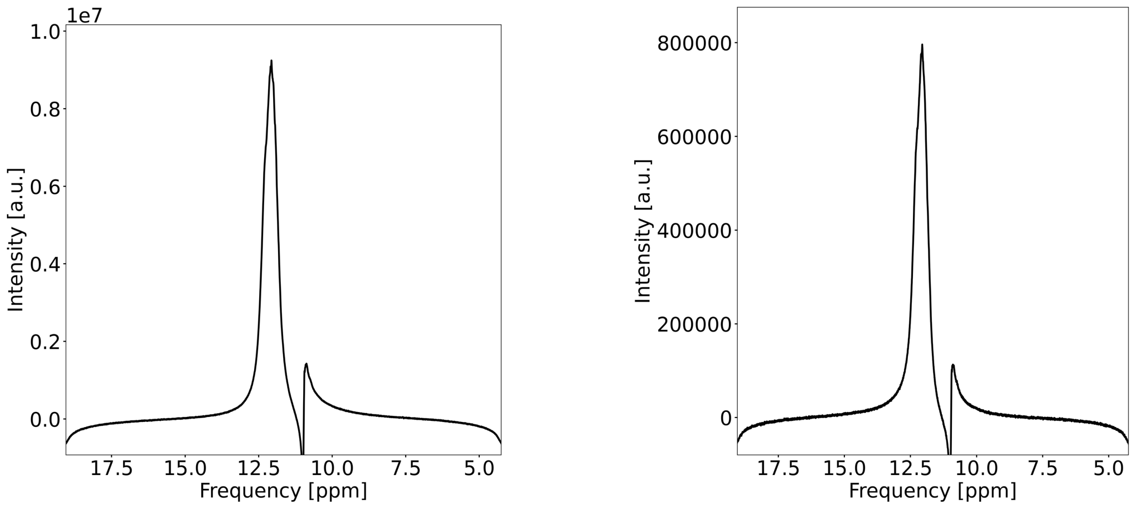

(a) (b)

Figure S24. Comparison of the ^{1}H NMR spectrum of the model setup for a QOC pulse optimized for $\Gamma_{B_1} = 1.2$ with a receiver gain of 1 (a) to a receiver gain of 36 (b). Hereby, the resonance at approx. 10.5 ppm is assigned to H_2O inside the copper cavity while the signal at approx. 12 ppm is assigned to H_2O in the PEEK cavity. The H_2O resonance in-between PEEK is effectively suppressed for both receiver gains.

S4.2.3 ν_1-selective QOC pulse performance dependence on shim

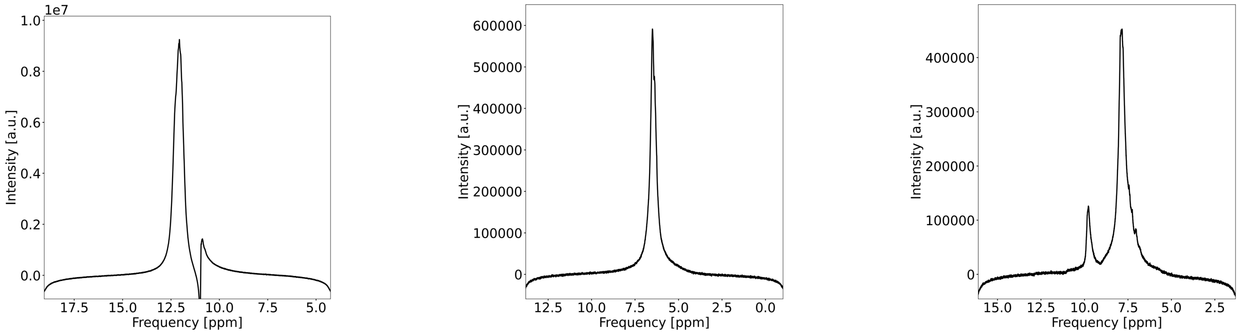

(a) (b) (c)

Figure S25. Comparison of the ^{1}H NMR spectrum of the model setup for a QOC pulse optimized for $\Gamma_{B_1} = 1.2$ with all shims set to zero (a), shimming until both resonances coalesce to a single resonance (b) and shimming to switch the frequencies of both resonances (c). Hereby, the resonance in (a) at approx. 10.5 ppm is assigned to H_2O inside the copper cavity while the signal at approx. 12 ppm is assigned to H_2O in the PEEK cavity. The resonance in (c) at approx. 7.5 ppm is assigned to H_2O inside the copper cavity while the signal at approx. 9.5 ppm is assigned to H_2O in the PEEK cavity. The H_2O resonance in-between PEEK is effectively suppressed in the case of all investigated shims.

S4.2.4 ν_1-selective QOC pulse performance dependence on $\Delta\nu_0$

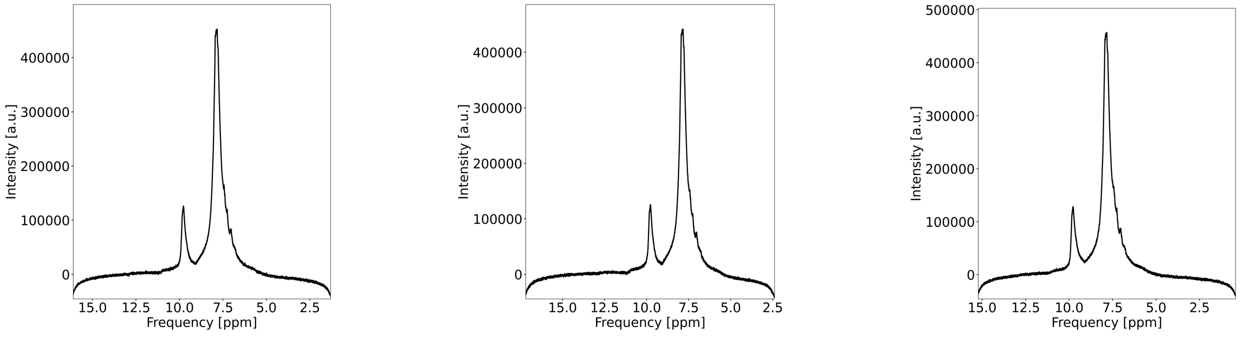

(a) (b) (c)

Figure S26. Comparison of the ^{1}H NMR spectrum of the model setup for a QOC pulse optimized for $\Gamma_{B_1} = 1.2$ with $\Delta\nu_0$ given in Section 2.2 (a), when moving $\Delta\nu_0$ downfield (b) and when moving $\Delta\nu_0$ upfield (c). Hereby, the resonance in (a) at approx. 7.5 ppm is assigned to H_2O inside the copper cavity while the signal at approx. 9.5 ppm is assigned to H_2O in the PEEK cavity. The H_2O resonance in-between PEEK is effectively suppressed in the case of all investigated $\Delta\nu_0$.

S4.3 Larmor frequency selective QOC pulse performance

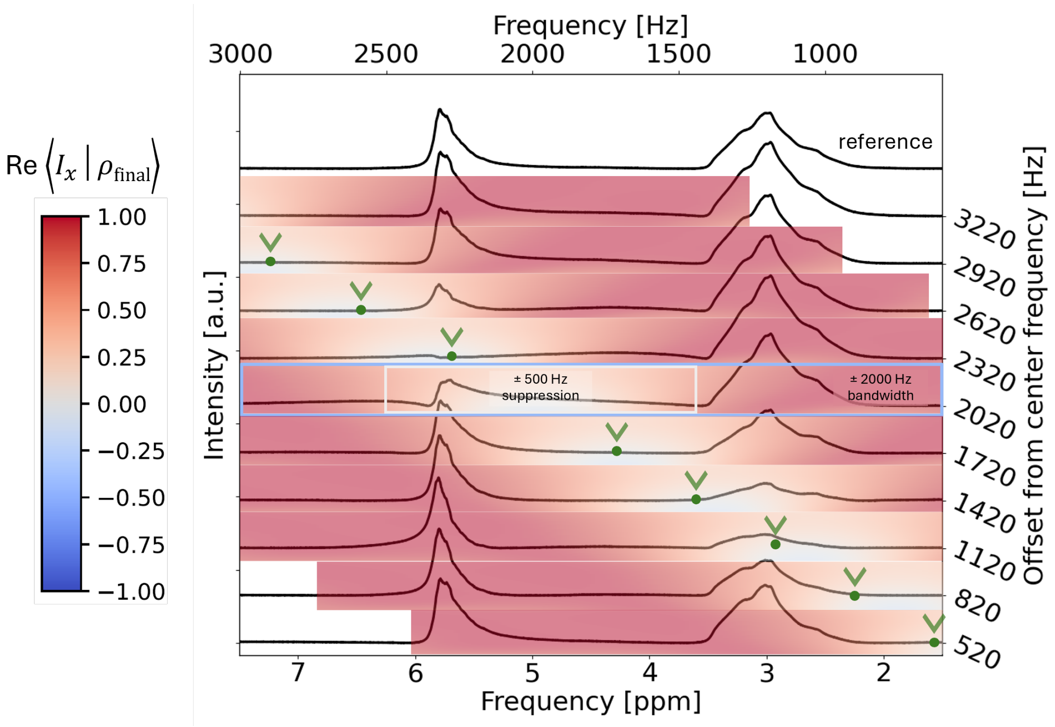

Figure S27. ^{1}H spectra recorded utilizing a ν_0-selective suppression pulse with a selective suppression range of 2.5 ppm (±500 Hz), 1 ms duration, and a total frequency range of 4000 Hz applied at different $\Delta\nu_0$. The top spectrum depicts the reference ^{1}H spectrum recorded using a hard pulse. Hereby, the resonance at approx. 3 ppm is assigned to n-dodecane and the resonance at approx. 6 ppm to H_2O. The spectra recorded with QOC pulses are underlaid with colour gradients representing the theoretical x-magnetization $\mathrm{Re}\,\langle I_x|\rho_{\mathrm{final}}\rangle$ after applying the QOC pulse at each particular $\Delta\nu_0$. Selective suppression is achieved for the on-resonance pulse with $\Delta\nu_0 = 2324$ Hz for H_2O. The pulse for n-dodecane is slightly off-resonance (+ 100 Hz) with $\Delta\nu_0 = 1424$ Hz and does not achieve sufficent suppression.

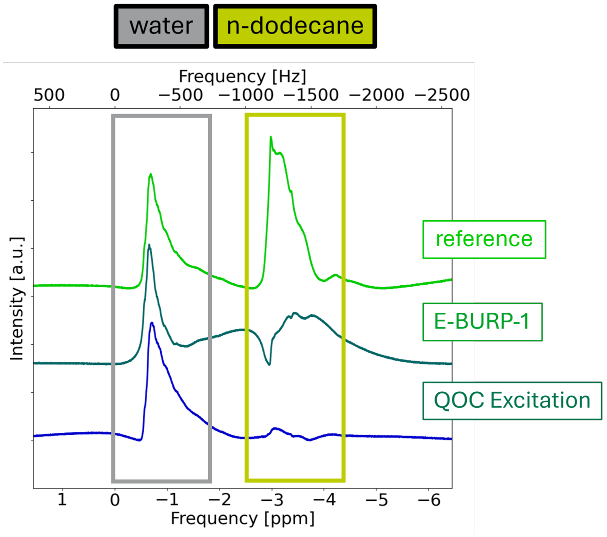

(a)

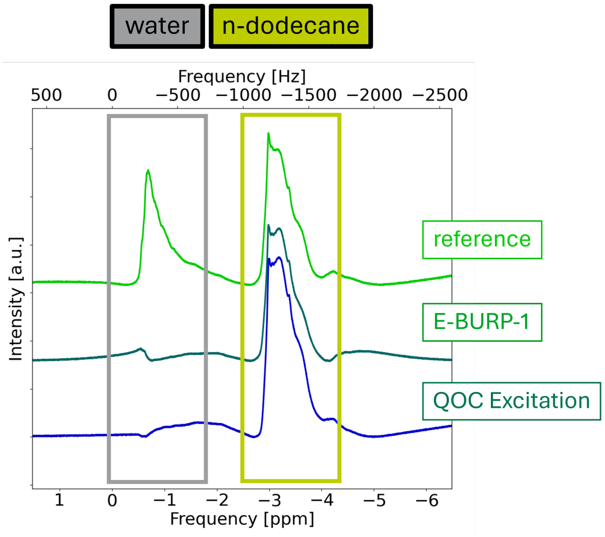

(b)

Figure S28. ^{1}H spectra recorded utilizing a ν_0-selective QOC excitation pulse (1 ms) versus an E-BURP-1 pulse (4 ms) with a selective excitation range of 2.5 ppm (±500 Hz). The top spectrum depicts the reference ^{1}H spectrum recorded using a hard pulse. Hereby, the resonance at approx. -3.3 ppm is assigned to *n*-dodecane and the resonance at approx. -0.8 ppm to H_2O. The pulses were applied on-resonance for either H_2O (a) or *n*-dodecane (b). The selective excitation of *n*-dodecane is achieved to a similar degree with both pulses, while the selective excitation of water is less efficient when using the E-BURP-1 pulse. The E-BURP-1 pulse is observed to distort the spectrum baseline and excites the *n*-dodecane resonance to a significant degree. The shift in frequencies with respect to previous measurements (other figures) originates from a drift of the magnetic field.

S5 Supporting data

S5.1 Individual relative integrals of the B_0-selective QOC pulses

Table S1. Individual relative integrals of the ν_0-selective 1 ms excitation pulses compared to a corresponding 90° hard pulse

$\Delta\nu_0$ [Hz]	H_2O excitation [%]	*n*-dodecane excitation [%]
2975	66.58 ± 0.41	0.90 ± 0.26
2775	95.15 ± 0.38	7.56 ± 0.24
2575	87.39 ± 0.36	11.56 ± 0.23
2375	87.45 ± 0.43	4.96 ± 0.28
2175	97.18 ± 0.38	9.71 ± 0.24
1975	95.03 ± 0.36	34.30 ± 0.23
1775	70.80 ± 0.37	63.17 ± 0.24
1575	35.81 ± 0.40	95.45 ± 0.26
1375	4.02 ± 0.40	115.34 ± 0.26
1175	13.58 ± 0.40	139.11 ± 0.26
975	14.40 ± 0.37	77.78 ± 0.24
775	8.89 ± 0.46	59.63 ± 0.30

Table S2. Individual relative integrals of the ν_0-selective 1 ms suppresion pulses compared to a corresponding 90° hard pulse. For this experiment, the baseline of the hard pulse spectrum required a comparatively strong correction, resulting in reduced integrals of the reference spectrum and thus increased relative integrals for all other spectra.

$\Delta\nu_0$ [Hz]	H_2O excitation [%]	*n*-dodecane excitation [%]
3224	175.34 ± 0.99	147.67 ± 0.31
2924	138.48 ± 1.41	134.16 ± 0.44
2624	57.89 ± 0.81	143.20 ± 0.25
2324	7.42 ± 0.97	159.24 ± 0.30
2024	65.17 ± 1.07	138.80 ± 0.33
1724	139.83 ± 1.09	88.81 ± 0.34
1424	173.06 ± 1.03	32.66 ± 0.32
1124	167.19 ± 0.94	25.24 ± 0.29
824	168.55 ± 1.11	68.70 ± 0.35
524	213.95 ± 0.99	110.54 ± 0.31

Table S3. Individual relative integrals of the ν_0-selective 2 ms suppresion pulses compared to a corresponding 90° hard pulse

$\Delta\nu_0$ [Hz]	H_2O excitation [%]	*n*-dodecane excitation [%]
2975	86.27 ± 0.46	99.92 ± 0.28
2775	40.62 ± 0.43	113.72 ± 0.26
2575	5.31 ± 0.41	93.40 ± 0.25
2375	1.01 ± 0.48	100.00 ± 0.30
2175	5.36 ± 0.45	90.79 ± 0.28
1975	34.54 ± 0.36	80.60 ± 0.22
1775	81.30 ± 0.43	51.14 ± 0.26
1575	102.77 ± 0.40	15.44 ± 0.24
1375	111.01 ± 0.42	1.29 ± 0.26
1175	119.03 ± 0.53	1.14 ± 0.33
975	103.97 ± 0.45	19.33 ± 0.28
775	99.13 ± 0.48	51.40 ± 0.30
